# A candidate gene analysis and GWAS for genes associated with maternal nondisjunction of chromosome 21

**DOI:** 10.1371/journal.pgen.1008414

**Published:** 2019-12-12

**Authors:** Jonathan M. Chernus, Emily G. Allen, Zhen Zeng, Eva R. Hoffman, Terry J. Hassold, Eleanor Feingold, Stephanie L. Sherman

**Affiliations:** 1 Department of Human Genetics, Graduate School of Public Health, University of Pittsburgh, Pittsburgh, Pennsylvania, United States of America; 2 Department of Human Genetics, Emory University School of Medicine, Atlanta, Georgia, United States of America; 3 Department of Biostatistics, Graduate School of Public Health, University of Pittsburgh, Pittsburgh, Pennsylvania, United States of America; 4 Center for Chromosome Stability, University of Copenhagen, Copenhagen, Denmark; 5 School of Molecular Biosciences and Center for Reproductive Biology, Washington State University, Pullman, Washington, United States of America; Stowers Institute for Medical Research, UNITED STATES

## Abstract

Human nondisjunction errors in oocytes are the leading cause of pregnancy loss, and for pregnancies that continue to term, the leading cause of intellectual disabilities and birth defects. For the first time, we have conducted a candidate gene and genome-wide association study to identify genes associated with maternal nondisjunction of chromosome 21 as a first step to understand predisposing factors. A total of 2,186 study participants were genotyped on the HumanOmniExpressExome-8v1-2 array. These participants included 749 live birth offspring with standard trisomy 21 and 1,437 parents. Genotypes from the parents and child were then used to identify mothers with nondisjunction errors derived in the oocyte and to establish the type of error (meiosis I or meiosis II). We performed a unique set of subgroup comparisons designed to leverage our previous work suggesting that the etiologies of meiosis I and meiosis II nondisjunction differ for trisomy 21. For the candidate gene analysis, we selected genes associated with chromosome dynamics early in meiosis and genes associated with human global recombination counts. Several candidate genes showed strong associations with maternal nondisjunction of chromosome 21, demonstrating that genetic variants associated with normal variation in meiotic processes can be risk factors for nondisjunction. The genome-wide analysis also suggested several new potentially associated loci, although follow-up studies using independent samples are required.

## Author summary

Approximately one of every 700 babies is born with trisomy 21—an extra copy of chromosome 21. Trisomy 21 is caused by the failure of chromosomes to segregate properly during meiosis, generally in the mother. Past studies have defined altered patterns of recombination along nondisjoined chromosomes as risk factors for human nondisjunction and model systems have clearly shown that specific genes involved recombination and other early meiotic processes play a role in the fidelity of chromosome segregation. However, no genome-wide genetic study (GWAS) has ever been conducted using maternal human nondisjunction as the disease phenotype. This study takes the first step to understand predisposing factors. We used chromosome 21 genotypes from the parents and child to identify mothers with nondisjunction errors derived in the oocyte and to establish the type of error (meiosis I or meiosis II). We then conducted a unique set of subgroup comparisons designed to leverage our previous work that shows that the etiologies of meiosis I and meiosis II nondisjunction differ for trisomy 21. Both the candidate gene study and the GWAS provide evidence that meiotic-specific structures and processes are vulnerable to genetic variants that lead to increased risk of human chromosome nondisjunction.

## Introduction

Correct segregation of chromosomes during the two successive meiotic divisions is essential for the formation of haploid gametes. At least 10% of human pregnancies produce aneuploid embryos with too many or too few chromosomes, the majority of which are lost during pregnancy. If they survive to term, many have severe congenital defects and developmental and intellectual disability. Thus, meiotic nondisjunction is the leading cause of pregnancy loss and birth defects in humans and an important limiting factor in women’s reproductive life span. (reviewed in [1–4]).

In humans, meiosis in females is highly prone to chromosome segregation errors [i.e., nondisjunction or premature separation of sister chromatids (PSSC)] and these errors increase exponentially with increasing maternal age. The differences between the development of oocytes and sperm clearly influence susceptibility for meiotic nondisjunction. Most importantly, they work on different timelines. In both sexes, meiosis starts with an initial step of DNA replication and the establishment of sister chromatid cohesion, followed by synapsis and recombination between homologous chromosomes. Homologs then separate at the end of meiosis I (MI), whereas sister chromatids separate in meiosis II (MII). Spermatogenesis begins after puberty and cells entering meiosis move from one stage to the other without delay. In contrast, oogenesis begins during fetal development and is arrested in prophase I after chromosomes synapse and recombine. MI resumes in the woman’s adult life just before the ovulation; MI is completed and the first polar body is extruded. MII begins but arrests for a short period as the oocyte travels through the Fallopian tubes, and is only completed if the oocyte is fertilized. Thus, meiosis in females extends over a 10 to 50 year period; the age of the woman at conception reflects the age of the oocyte, and basically the period of arrest in MI. Given the mechanistic differences and temporal separation of maternal MI and MII, it is not surprising that associated risk factors differ for MI and MII nondisjunction errors (reviewed in [5]).

Trisomy 21 has become an important resource to understand meiotic nondisjunction in humans, as it is one of the few aneuploid conditions that survives to term. However, even for trisomy 21, involving the smallest human autosome, about 50–80% conceptions are estimated to be lost during pregnancy [6, 7]. Using chromosome 21 genetic markers to categorize the type of meiotic error among live births with trisomy 21, over 90% are derived from errors in the oocyte, of which at least 75% are estimated to be initiated in MI and about 25% in MII (e.g., [8]).

In this study, our goal was to discover genetic variants that increase the risk for maternal nondisjunction of chromosome 21 using both a candidate gene approach and a genome-wide association study. We focused on candidate genes that have been associated with chromosome dynamics early in meiosis. Accurate segregation depends on the coordinated control of sister chromatid cohesion with chromosome synapsis and the assembly of the synaptonemal complex (SC) and, within these structures, meiotic recombination [9, 10]. Below we provide a brief overview of the role of some of the important meiotic genes that mediate these processes, and that we have examined in the present study. Bolcun-Filas and Schimenti [9] have summarized the meiotic defects that are observed in the associated mutant mouse models.

In a meiotic cell, DNA is organized as an array of loops along a proteinaceous axis. The axes are composed of the meiosis-specific synaptonemal complex, in association with condensin/cohesin complexes. Several of the components of meiotic cohesin are meiosis-specific, including those encoded by *SMC1β*, *REC8*, *RAD21L*, and *STAG3*. The SC brings homologous chromosomes into close proximity and promotes recombination and chiasmata formation [11]. The mature SC is a tripartite structure, composed of two parallel axial/lateral elements that bind to each homolog and a central element, with transverse filaments joining the individual axial/lateral elements [12, 13]. SYCP2 and SYCP3 are components of the axial/lateral elements. SYCP1 is a component of the transverse filaments and components of the central element are encoded by *SYCE1*, *SYCE2*, *SYCE3*, and *TEX12*. In addition to these structural sub-units, *HORMAD1* and *HORMAD2* code for proteins that load onto axes of meiotic chromosomes throughout early prophase I but are removed upon synapsis, a process that depends on the presence of TRIP13 [14]. In general, HORMAD1 and HORMAD2 play a role in coordinating progression of chromosome synapsis with meiotic recombination [15].

Meiotic recombination is initiated by programmed DNA double-strand breaks (DSBs) that occur as the meiotic chromosome axes develop early in prophase I. These breaks are generated by the SPO11 protein and its interacting partners MEI1, MEI4 and REC114 (reviewed in Cole et al. [16]). The DSBs are processed to generate single-stranded DNA that is bound by strand-exchange proteins DMC1 (meiosis specific) and RAD51 (ubiquitously expressed). The single-stranded DNA then engages in homology search. Proper function of DMC1 requires interactions with several meiotic accessory proteins, one of which is MND1. MND1, complexed with HOP2, stabilizes the DMC1 filaments on the resected end of the DSBs. This complex also increases the ability of the pre-synaptic filament to capture the double-stranded DNA (reviewed in Sansam and Pezza [17]).

As homologs synapse, so-called early recombination nodules transiently associate with ZMM proteins, including DNA mismatch repair proteins MSH4 and MSH5. Subsequently, a proportion of these are converted into late recombination nodules, detected by the mismatch repair proteins MLH1 and MLH3, and representing the vast majority of crossovers [18–22]. In addition, EXO1 and BLM function in crossover regulation, and with MLH1 and MLH3, appear to play a role in the crossover pathway that is subject to crossover interference (reviewed in Manhart and Alani [23]).

In addition to these candidate genes, we chose another group of genes that have been associated with the amount of global meiotic recombination in humans. The motivation for these candidates is based on the altered recombination patterns observed along nondisjoined chromosomes, a well-established predisposing factor for maternal nondisjunction of almost all human chromosomes studied to date (reviewed in [24]). Specifically for maternal chromosome 21 nondisjunction, altered meiotic recombination patterns are associated with both MI and MII error types [25–29]. For maternal MI-derived trisomy 21, about 40–47% of MI cases are derived from oocytes with no meiotic exchange [25, 27, 30]. Among those with a single exchange, the majority of exchanges occur in the telomeric region of chromosome 21. MII errors are associated with pericentromeric exchanges [25, 27, 29, 30]. This apparent effect of an MI process–recombination–on MII nondisjunction suggests that at least a portion of so-called MII errors may have their origin in MI. In addition, there is evidence that genome-wide recombination counts in oocytes with a MI nondisjunction error of chromosome 21 are reduced compared to oocytes with normal meiosis [31, 32]. Also, previous studies indicate that oocyte-specific dysregulation of global recombination may contribute to the nondisjunction event [31]. Thus, we chose candidate genes identified in the largest GWAS study of meiotic recombination conducted on humans, a study based on 71,929 parent-offspring pairs from Iceland [33]. They found evidence for 13 variants in eight regions that were associated with genome-wide recombination counts.

For both the candidate gene and genome-wide association studies, we took a unique approach by using several different GWAS group comparisons (Table 1). These comparisons were crafted to address the likelihood that there are both distinct genetic factors influencing MI and MII nondisjunction and common factors affecting both. In addition, some of our analyses target the conflated phenotype of nondisjunction with survival to term. Study design issues are discussed in more detail below.

**Table 1:** Description of primary analyses and associated sample sizes.

Analysis	Analysis groups	Sample size	Contrast able to detect:
Logistic regression	Mothers vs. fathers	705 vs. 645	Maternal NDJ and survival to term
. . .	MI mothers vs. fathers	535 vs. 645	MI-specific effects and survival to term
. . .	MII mothers vs. fathers	157 vs. 645	MII- specific effects and survival to term
. . .	MI mothers vs. MII mothers	535 vs. 157	MI- or MII-specific effects
TDT	All complete case trios	615 trios	Survival to term

NDJ: nondisjunction; MI: maternal MI nondisjunction error; MII: maternal MII nondisjunction error.

## Methods

### Study sample

Our study participants included 749 live born offspring with free (non-translocation), maternally-derived trisomy 21 (both full and mosaic trisomy 21 were included) and their available biological parents. In almost all instances, the trisomy was confirmed by karyotype, although in some it was confirmed by birth record or parent report. Recruitment occurred in the U.S. by multiple sites since 1989, when the first population-based study was initiated. Recruitment for these population-based studies used birth surveillance systems to identify infants born with Down syndrome (details are provided in Freeman et al. [8]). Later, our recruitment strategy was not population-based, but instead a convenience sample of families with Down syndrome identified through our network of assessment sites, social and website media, and parent groups. Using self-reported race/ethnicity, 72% reported as White, 4% as Hispanic descent, 2% as African/African-American or Asian descent and about 23% with other or unknown descent.

### Ethics statement

Participants were recruited from several geographic areas with the collaboration of several institutions, including Arkansas (University of Arkansas for Medical Sciences, Arkansas Center for Birth Defects Research and Prevention, Arkansas Children’s Hospital, Arkansas Reproductive Health Monitoring Systems), California (California Birth Defects Monitoring Program, Public Health Institute), Georgia (Department of Human Genetics, Emory University; Centers for Disease Control and Prevention), Iowa (University of Iowa, Registry for Congenital and Inherited Disorders), New Jersey (New Jersey Department of Health and Senior Services; Special Child Health Services Registry; Eagleton Institute), and New York (New York State Department of Health Congenital Malformations Registry). Each recruitment site obtained IRB approval for their protocol, consent forms, and data sharing during the project period from their respective institutions. All samples were collected under written consent by each participant or their legal guardian. Emory University was the site for the data and biorepository. They obtained IRB approval for all sample processing and de-identified sample submission to the Center for Inherited Disease Research genotyping service (Emory School of Medicine IRB number IRB00005100). IRB approvals for genotyping samples and uploading to dbGaP were approved prior to the initiation of that genotyping project (dbGaP: phs000718).

### Genotyping

DNA samples were obtained from lymphoblastoid cell lines (LCLs) (36.8%), saliva (23.7%), buffy coat (15.7%), whole blood (13%), unknown source (i.e., no record available) (8.4%), and buccal cell (0.09%). The remaining 2.2% of genotyped samples were HapMap controls derived from LCLs that were used by the Center for Inherited Disease Research (CIDR) for quality control (QC). The samples were genotyped in batches corresponding to 96-well plates and each plate contained two study duplicates and HapMap controls. Duplicates were randomly selected from all samples with sufficient DNA. Families were randomly distributed across plates with all members of each family on the same plate.

Genotyping was performed on the Illumina HumanOmniExpressExome-8v1-2 array by the Center for Inherited Disease Research (CIDR). The algorithm used for calling genotypes was GenomeStudio version 2011.1, Genotyping Module version 1.9.4 and GenTrain version 1.0. Genotype data that passed initial QC at CIDR were released to the Quality Assurance (QA)/QC) analysis team at the University of Washington Genetics Coordinating Center (UWGCC) for data cleaning and imputation. Details of these procedures can be found in Laurie et al. [34] and all data are available in dbGaP along with specifics of genotyping and QC (dbGaP: phs000718). After QC, genotypes were available for 2,186 unique study participants. We filtered SNPs based a deviation of Hardy-Weinberg equilibrium (HWE) at p < 10^−6^. Overall, the median call rate was 99.86% and the error rate estimated from 53 pairs of study sample duplicates is 1x10^-4^. All samples had a missing call rate < 2%. The percent of SNPs with a minor allele frequency (MAF) of < 2% was 30% for the autosomes and 32.1% for the X chromosome. This calculation was based on all study participants for SNPs not located on chromosome 21 and on only study parent samples for SNPs on chromosome 21. Trisomic genotypes for all 749 children in the study were called from raw genotyping data with previously-developed methods [35].

Possible chromosomal abnormalities beyond trisomy 21 were examined as possible artifacts of the use of DNA from LCLs. This was done using Log R Ratio" (LRR) and “B Allele Frequency" (BAF) [36, 37] and applying the methods outlined in Laurie et al. [38]. Regions or chromosomes containing identified anomalies were excluded for genotype imputation purposes (see below). For chromosomes other than chromosome 21, 50 large anomalies were identified, of which 15 were filtered out of the dataset by setting genotypes in the identified region to missing. In addition, Mendelian inconsistencies were examined and one additional family was identified to have a genotype pattern consistent with uniparental chromosome 16 in the offspring. Genotypes at this chromosome were also set to missing.

Seven participants with neither parent genotyped were excluded from subsequent analyses. Thus in the remaining 742 families, genotypes were available for both the child and either the mother only (n = 95), the father only (n = 17), or both parents (n = 630).

### Adjustment for population structure

Binary trait analyses using logistic regression are our primary statistical approach in this GWAS study. To adjust for population structure, we first used principal components analysis (PCA) as described by Patterson et al. [39], and implemented in R (SNPRelate package). SNPs used for PCA were selected by LD pruning from an initial pool that included all non-chromosome 21 autosomal SNPs with a missing call rate < 5% and MAF > 5%. In addition, the 2q21 (LCT), HLA, 8p23, and 17q21.31 regions were excluded from the initial pool. The first three eigenvectors were used in subsequent analyses.

### Imputation

The UWGCC used IMPUTE2 software [40] to perform genotype imputation. Details of their methods and QC are available at dbGaP:phs000718. The worldwide reference panel of 1,092 samples from the 1000 Genomes Project’s Phase I integrated variant set [41] was used for imputation. We included only imputed variants with a quality metric of ≥ 0.3, as previously recommended [42].

### Phenotyping

Our primary association studies were based on mothers who had a live birth with full or mosaic trisomy 21 as determined by karyotype and then determined to be due to a maternal nondisjunction error based on the characterization of the chromosome 21 genotype contributions from parent to the child with trisomy 21. Genotypes were obtained from the Illumina HumanOmniExpressExome-8v1-2 array and from previously genotyped variants along chromosome 21 using both STRs and SNPs [27, 29] The groups based on maternal nondisjunction errors were compared with fathers of the children with trisomy 21 who represent a random group of individuals from the population.

Methods for defining the type of nondisjunction errors are described in detail in our previous work (e.g.,[27, 29]). Briefly, parental origin of the meiotic error (maternal or paternal) was first determined by establishing the contribution of informative parental chromosome 21 genotypes to the child with trisomy 21. In families with both parents genotyped and where the parent of origin was unambiguously confirmed to be the mother (the vast majority of these families), we defined the meiotic stage of origin. We scored the genotype at each informative SNP and STR marker on chromosome 21q as either reduced to homozygosity (R) or not (N), according to whether the mother transmitted two identical or two different alleles, respectively, to her child at that locus. The meiotic stage of nondisjunction (MI or MII) was called according to the zygosity at the loci most proximal to the centromere (N or R, respectively). In a few cases (n = 7), MII nondisjunction was called on the basis of a single, well-genotyped R SNP nearest the centromere (followed by a series of N SNPs), but because of the dense SNP genotyping on the chip, stage was more typically supported by many markers.

In families with only one parent genotyped, a slightly different approach was required, as missing parental data led to some markers that are partially informative, but not dispositive of zygosity. Briefly, we considered the ratio of information in the SNPs near the centromere, and called each case as MI or MII depending on the ratio. The threshold for this ratio was selected by performing an experiment with the complete trios; for each complete trio, we masked the genotype of one parent, calculated the ratio described above, and found the cutoff that optimized the predictive accuracy.

Lastly, when all informative markers in the parent of origin were reduced to homozygosity along 21q, the origin of error was inferred to be a post-zygotic, mitotic error and the case was excluded from this study, consistent with previous studies [25]. However, we recognize that a proportion of these cases may be MII nondisjunction errors with no recombination.

### Analysis

#### Sample size

As described above, samples from 2,186 participants were genotyped for this study, comprising 749 children and 1,437 parents. Participants with unresolved identity swaps, probands (children) with neither parent genotyped, and mothers in cases of potentially mitotically-arising trisomy were excluded from GWAS. After this filtering, 705 mothers and 645 fathers were retained for analysis, comprising 612 complete parent-child trios. Meiotic stage of origin for trisomy was determined to be MI in 535 cases, MII in 157 cases, and was not determined in 13 cases. Sample sizes for our analysis groups are reported in Table 1.

#### Association studies

We performed five primary GWAS analyses (summarized in Table 1). The comparison for all mothers vs. fathers can identify maternal genetic factors influencing nondisjunction either in MI or MII (or, more powerfully, in both). As noted in the Introduction, some genetic factors affecting MI nondisjunction may be shared with MII nondisjunction. Comparison of MI-only or MII-only mothers with fathers can identify maternal genetic factors influencing MI nondisjunction or MII nondisjunction, respectively. All three of these comparisons will also detect maternally-derived variants affecting survival of the infant to term. We chose to use fathers within our own study as controls rather than turning to external controls because of the significant problem with confounding (chip and study effects) that is introduced when cases genotyped in one study are compared to controls genotyped in another.

However, one risk of using fathers as controls is that in theory the three analyses that compare mothers to fathers may also identify spurious associations due to comparing females to males. We tested this by running a female vs. male GWAS in a large additional dataset and comparing our results to those. The dataset we used was a subset of the COHRA study [43]; this study targeted dental phenotypes, but participants were selected in a community-based setting without regard to phenotype. We used 456 male and 494 female unrelated self-identified white adults in order to have a sample size comparable to the current study. By using sex as the outcome measure in a sample that was unselected with regard to phenotype, this analysis gave us a set of results to compare to our trisomy dataset in order to determine whether any of our trisomy results might instead be male vs. female artifacts. The female vs. male analysis in the COHRA dataset did not result in any unusually significant differences (lambda = 0.94). None of the GWAS loci or candidate genes described in the results section appeared among the largest differences between males and females in the COHRA dataset. The Manhattan plot and QQ plots are provided in the Supporting Information (S1 Fig), as well as results from the COHRA analysis in our candidate genes (S1 Table).

The fourth comparison involves MI vs. MII mothers. This comparison has the potential to identify unique factors for MI or MII nondisjunction without confounding by trisomy 21 survival; that is, both groups of mothers had a live birth child with trisomy 21.

For the fifth analysis, we conducted a transmission disequilibrium test (TDT) [44]. This test examines the association between the *child’s* genotype and the dual phenotype of nondisjunction and survival to term. Our prior hypothesis is that this test is best for identifying fetal “survival genes.” If there is association between *maternal* genotype and either nondisjunction or survival, this test can in theory identify it, but the association would be weakened. We did not perform this test for the candidate genes, since they were chosen as candidates for nondisjunction, not for survival. For the nondisjoined chromosome 21, we used a trisomic TDT, previously developed by our group [45].

For all analyses except the TDT, we used the logistic regression model *logit(p) = SNP + PC1 + PC2 + PC3*, where *SNP* is encoded additively and *PC1*, *PC2*, and *PC3* are the first three principal components of ancestry. The X chromosome was not examined because our primary comparative analyses involved mothers vs. fathers.

For all analyses, we filtered out SNPs with MAF < 1% or with extreme departure from HWE. Imputed SNPs with info score < 0.5 were also excluded, and imputed genotypes called with less than 90% confidence were coded as missing. Analyses were performed with PLINK and R.

#### Maternal age effect

Because of the strong maternal age effect in maternal chromosome 21 nondisjunction, it is important to consider how maternal age fits into the above analyses. Previous results from our group and others suggest not only different etiologies for MI and MII nondisjunction, but likely different etiologies in different age groups. Statistically, this would suggest a model that includes not only maternal age effect but also an age X genotype interaction term. However, since our primary analyses compare mothers to fathers, it is not possible to fit such a model (since fathers have no “maternal age”). The logical analysis, then, is to stratify by maternal age group, similar to the approach we took for the MI and MII subgroups. We performed several such analyses, but the sample sizes were prohibitively small for interpretation. We elaborate further on this issue in the Discussion.

#### Candidate gene analyses

For candidate gene analyses, we examined a window of 60kb on each side of the gene or SNP. We used a statistical significance cutoff based on the method of Li and Ji [46], which calculates the equivalent number of independent SNPs in the region and applies a Bonferroni correction based on that number. Thus the candidate gene analyses are fully corrected for multiple testing at the level of each individual gene.

#### Follow-up analyses to examine top-ranked GWAS signals

For follow-up analyses of signals of p < 10^−5^ for the GWAS, we performed literature searches on genes within 500kb. For each of those regions, LocusZoom plots were created in all analyses to identify common associations across analyses.

## Results

### Candidate gene association studies

**Fig 1:**
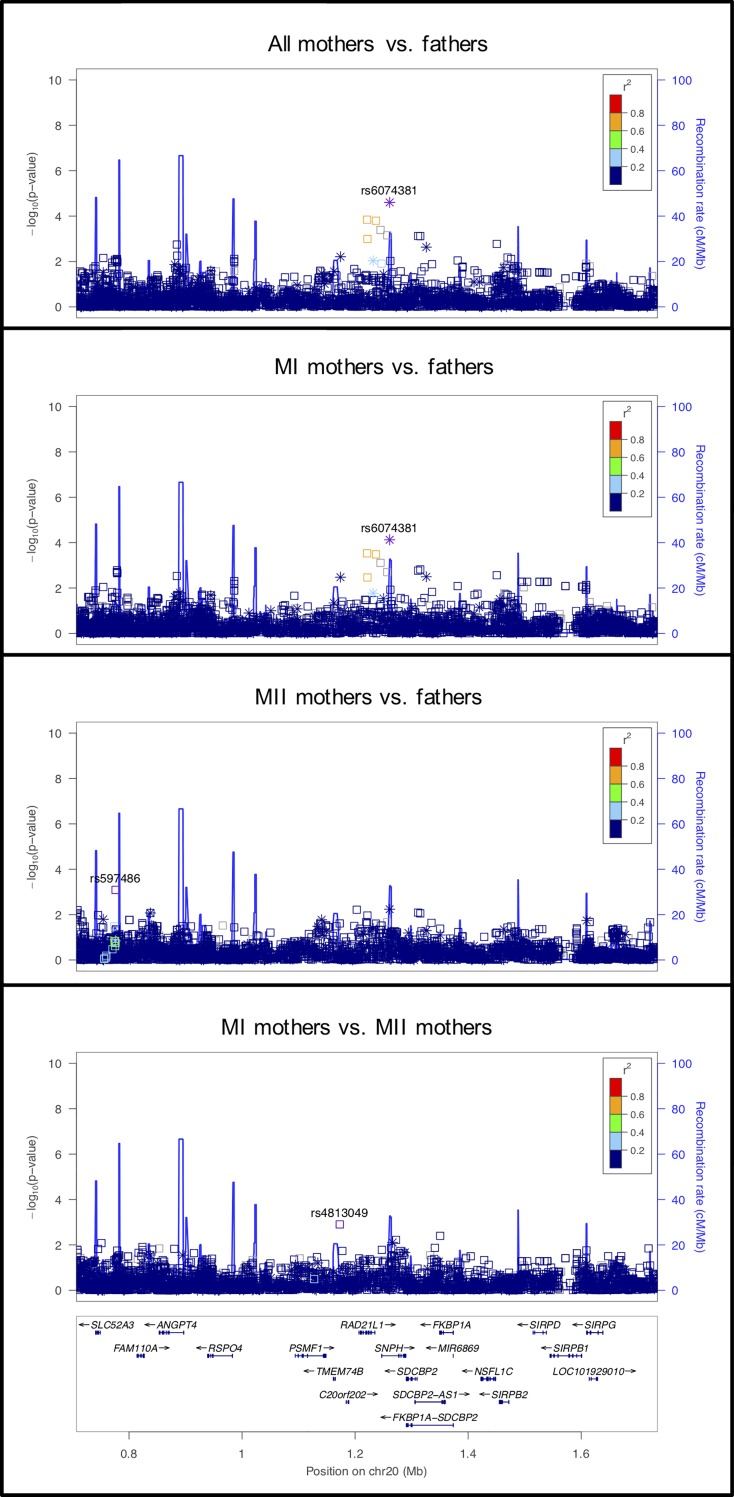
LocusZoom plot for *RAD21L*. In this Figure (as in Figs 2–12) four LocusZoom plots show the results at one locus across all four analyses. Each point is one variant, with the *x*- and *y*-axes representing physical position on the chromosome and -log_10_(p-value), respectively. Open squares and asterisks represent genotyped and imputed variants, respectively. Coloring represents linkage disequilibrium (red = stronger, blue = weaker) with the tagging SNP (which is purple). The overlaid blue curve shows the recombination rate.

**Fig 2:**
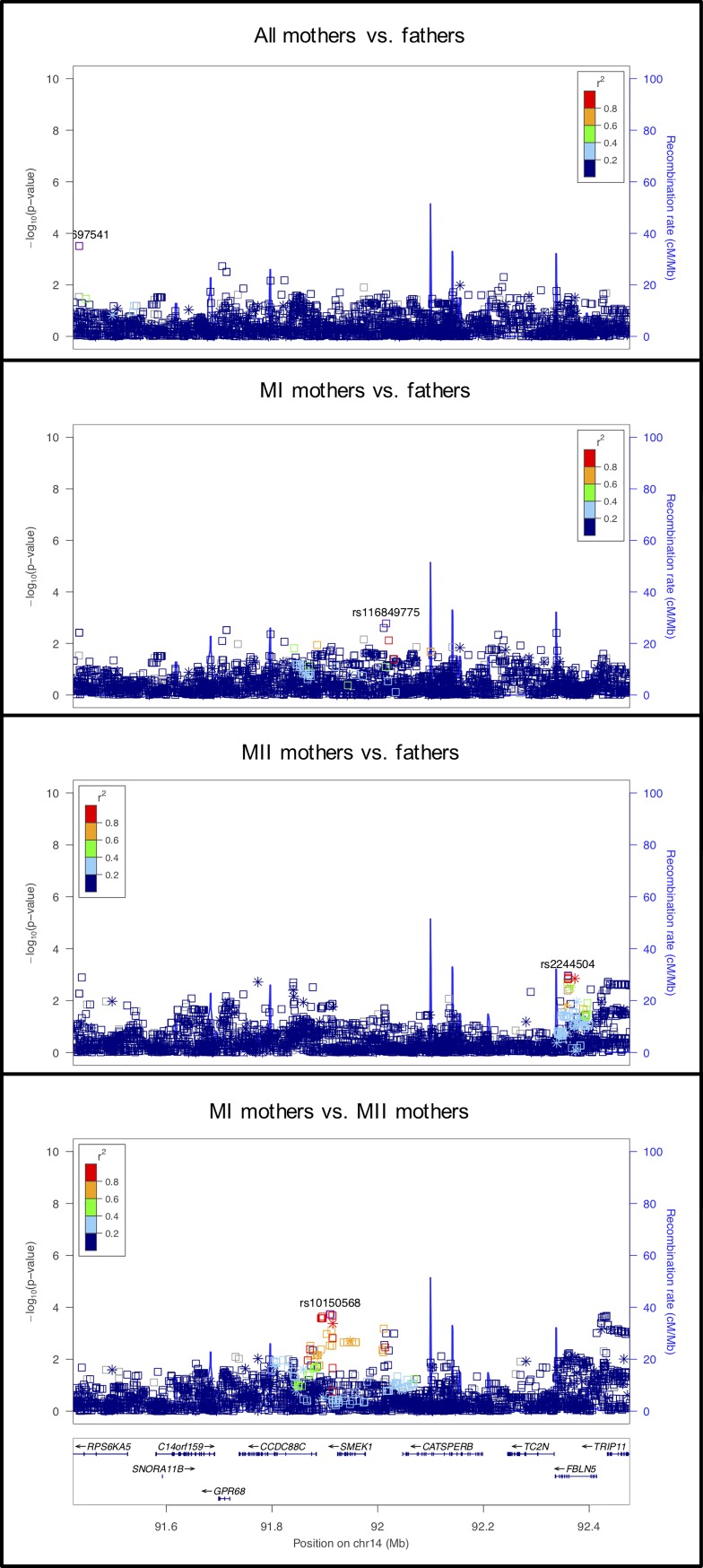
LocusZoom plot for *SYCE2*.

**Fig 3:**
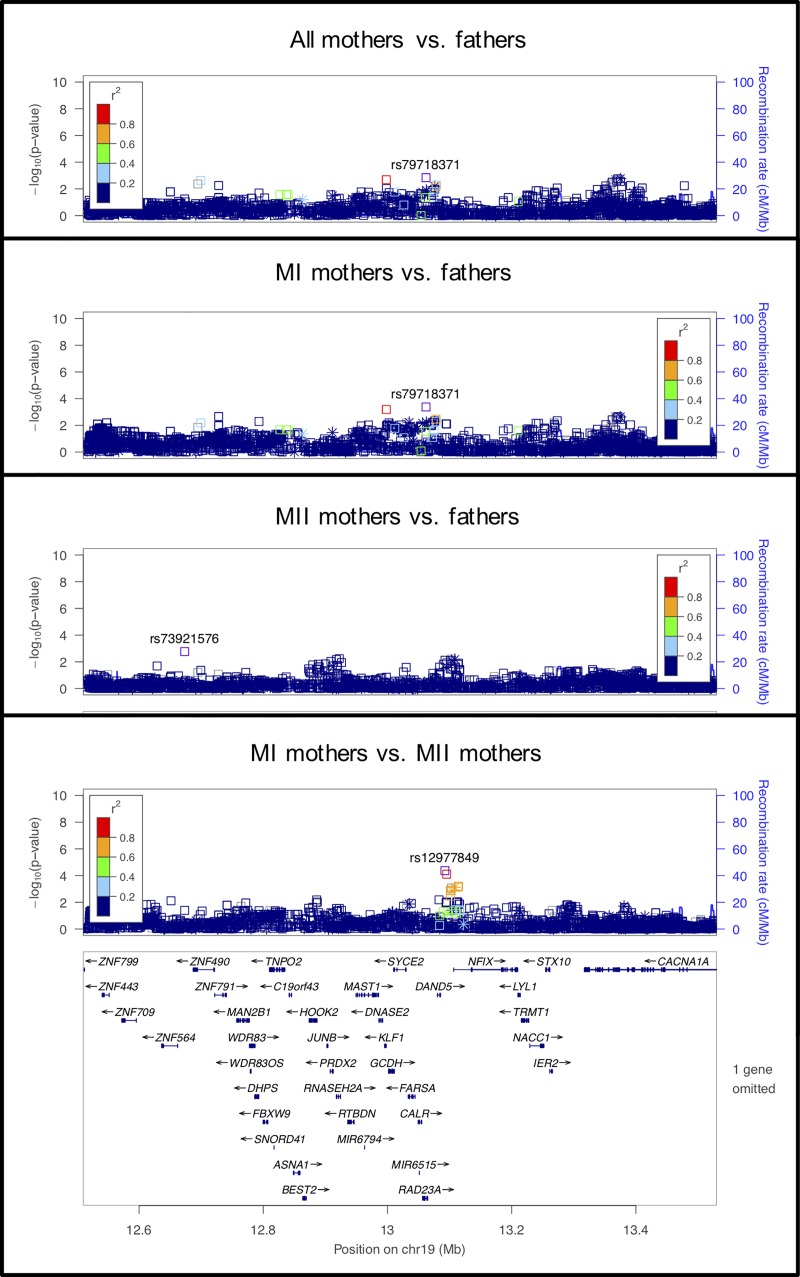
LocusZoom plot for *SYCP1*.

**Fig 4:**
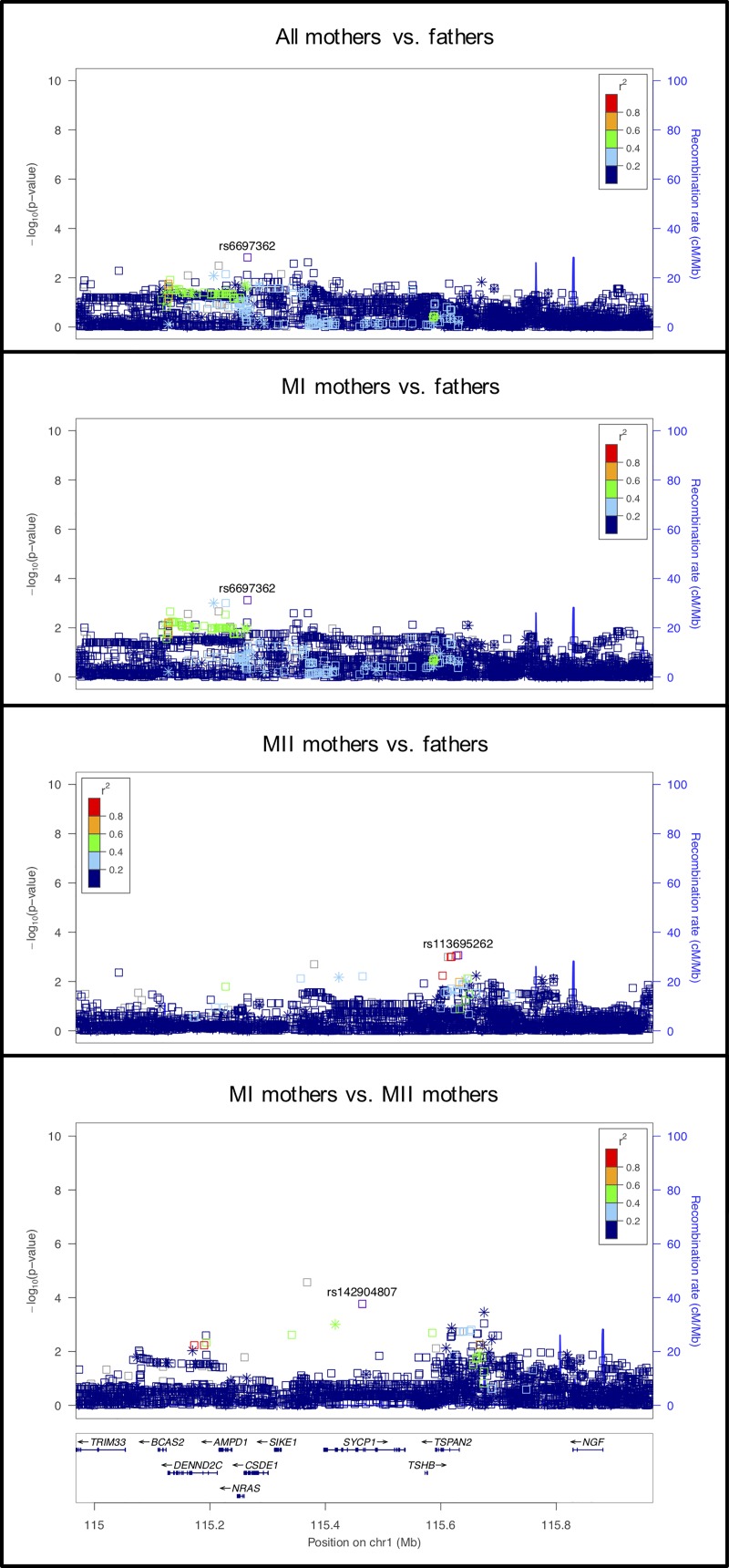
LocusZoom plot for *SYCP2*.

**Fig 5:**
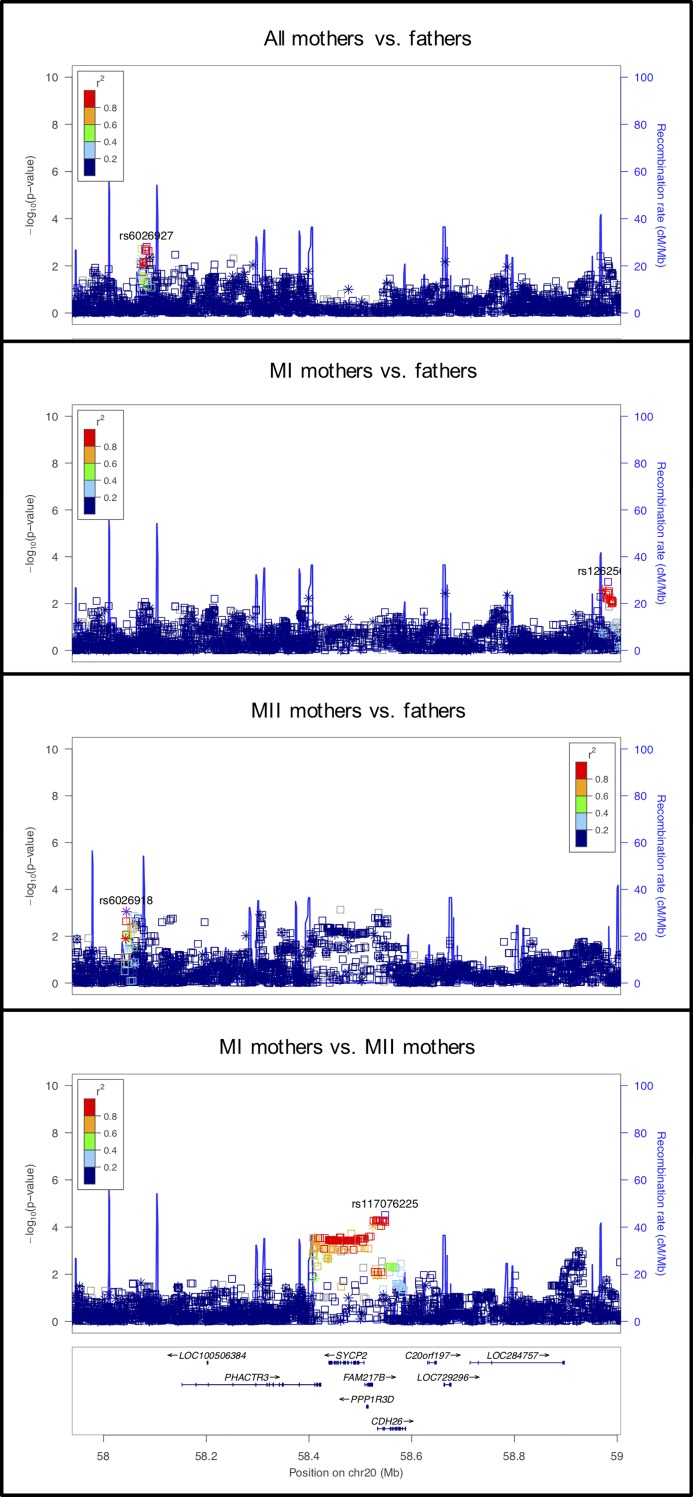
LocusZoom plot for *SMEK1*.

We focused on two sets of candidate genes/regions: genes that function in early stages of meiosis and that have been associated with accurate chromosome segregation (n = 24) and regions associated with human recombination genome-wide counts (n = 8) [33]. The Bonferroni-corrected statistical significance cutoffs along with all results are shown in Table 2 and LocusZoom plots are provided in Figs 1–5 and in the Supporting Information (S2–S9 Figs). Each row in Table 2 represents one candidate locus. Each column represents an analysis. For each cell in the table, the most significant association at the locus (not always unique) is reported. P-values significant after correcting for multiple testing are marked with an asterisk and highlighted. Note that for each analysis in each gene, Table 2 lists the most statistically significant result, so that the SNP that appears in a given gene is not necessarily the same in each analysis. More detailed results are shown in the Supporting Information (S2 Table).

**Table 2:** Candidate gene results.

Locus	All mothers vs. fathers	MI mothers vs. fathers	MII mothers vs. fathers	MI mothers vs. MII mothers	Significance threshold
*SYCP1*	P = 0.00238	P = 0.00255	P = 0.002	P = 2.69e-05*	9.43e-04
*SYCP2*	P = 0.017	P = 0.00592	P = 0.000735*	P = 3.09e-05*	8.62e-04
*SYCP3*	P = 0.0078	P = 0.00748	P = 0.00637	P = 0.00151	9.43e-04
*SYCE1*	P = 0.0336	P = 0.022	P = 0.0227	P = 0.00222	1.35e-03
*SYCE2*	P = 0.00146	P = 0.000425*	P = 0.0154	P = 0.00655	1.14e-03
*SYCE3*	P = 0.00324	P = 0.000764	P = 0.0053518	P = 0.00337	5.38e-04
*TEX12*	P = 0.00558	P = 0.0219	P = 0.013871	P = 0.0271	1.19e-03
*BLM*	P = 0.0225	P = 0.0378	P = 0.00601	P = 0.0257	7.04e-04
*DMC1*	P = 0.0184	P = 0.00993	P = 0.018619	P = 0.0134	1.19e-03
*EXO1*	P = 0.00924	P = 0.0118	P = 0.00111	P = 0.00303	6.10e-04
*HORMAD1*	P = 0.0152	P = 0.0174	P = 0.011377	P = 0.00361	1.35e-03
*HORMAD2*	P = 0.0113	P = 0.0156	P = 0.00155	P = 0.00838	8.93e-04
*MEI1*	P = 0.00655	P = 0.0485	P = 0.00951	P = 0.0204	9.26e-04
*MEI4*	P = 0.0147	P = 0.0164	P = 0.0283	P = 0.021	6.33e-04
*MLH1*	P = 0.0151	P = 0.00297	P = 0.0162	P = 0.0427	1.14e-03
*MLH3*	P = 0.00472	P = 0.0107	P = 0.0241	P = 0.0207	1.35e-03
*MND1*	P = 0.0273	P = 0.013	P = 0.000336*	P = 9.6e-05*	8.77e-04
*MSH5*	P = 0.00677	P = 0.00135	P = 0.0417	P = 0.0241	9.26e-04
*REC114*	P = 0.0187	P = 0.0272	P = 0.0886	P = 0.0118	8.77e-04
*REC8*	P = 0.00822	P = 0.00404	P = 0.00195	P = 0.00112	9.43e-04
*SMC1B*	P = 0.0115	P = 0.00407	P = 0.047	P = 0.0349	7.81e-04
*SPO11*	P = 0.0232	P = 0.0325	P = 0.0115	P = 0.0181	1.25e-03
*STAG3*	P = 0.005	P = 0.00472	P = 0.0798	P = 0.00958	1.19e-03
*TRIP13*	P = 0.0026	P = 0.0122	P = 0.00358	P = 0.0206	1.61e-03
*rs1254319* (C14orf39 missense)	P = 0.0278	P = 0.0488	P = 0.0372	P = 0.00464	1.39e-03
*rs75502650* (CCDC43 intron)	P = 0.00203	P = 0.00137*	P = 0.000628*	P = 0.031	7.58e-04
*rs1132644* (CCNB1IP1 UTR)	P = 0.0232	P = 0.00657	P = 0.0236	P = 0.0195	1.32e-03
*rs56162163* (chr17 inversion)	P = 0.00666	P = 0.00956	P = 0.00737	P = 0.0166	6.67e-04
*rs74434767* (CPLX1 intron)	P = 0.00611	P = 0.0178	P = 0.0227	P = 0.0329	1.61e-03
*rs5745459* (MSH4 missense)	P = 0.0389	P = 0.0283	P = 0.037	P = 0.0198	1.16e-03
*rs150798754* (PRDM9 intergenic)	P = 0.00219	P = 0.006	P = 0.0123	P = 0.022	1.25e-03
*rs6889665* (PRDM9 upstream)	P = 0.00219	P = 0.006	P = 0.0104	P = 0.0678	8.33e-04
*rs450739* (RAD21L missense)	P = 2.49e-05*	P = 7.47e-05*	P = 0.00579	P = 0.00126	1.09e-03
*rs4045481* (RNF212 missense)	P = 0.00475	P = 0.0136	P = 0.00514	P = 0.0131	1.02e-03
*rs658846* (RNF212 intron)	P = 0.00292	P = 0.0108	P = 0.00514	P = 0.0131	8.33e-04
*rs12233733* (RNF212 nearby)	P = 0.00292	P = 0.0108	P = 0.00396	P = 0.031	1.52e-03
*rs10135595* (SMEK1 UTR)	P = 0.0125	P = 0.007	P = 0.00306	P = 0.000183*	1.39e-03

Each row represents one candidate locus (either a gene with a 60kb border on each side or a 60kb window around a SNP). Each column represents an analysis. For each locus-analysis pair, the most significant association at the locus (not always unique) is reported. P-values significant after correcting for multiple testing (i.e., exceeding the Bonferroni-adjusted significance threshold noted in the last column) are marked with an asterisk and highlighted. (MI: meiosis I; MII: meiosis II; P: p-value; OR: odds ratio.) The first 24 loci represent genes selected for their function (above the double line). The latter 13 loci represent SNPs identified by Kong et al. in their GWAS of recombination [33], with annotation in parentheses (below the double line).

#### Candidate genes associated with chromosome segregation

These genes are shown in the top half (above the double line) of Table 2. Examination of genes involved in the meiotic cohesion complex showed a statistically significant association with *RAD21L*, a meiosis-specific member of the α-kleisin protein family [47–50]. This was significant in both the mothers vs. fathers and the MI mothers vs. fathers comparisons, and has a similar effect (odds ratio) in the MII cases at the same SNP (Fig 1). Meiotic cohesins are essential for sister chromatid cohesion, but also have an effect on other prophase I processes, including formation of the axial/lateral elements, assembly of the SC, and crossing-over (e.g., [51, 52]). Gene disruption of RAD21L leads to sexually dimorphic phenotypes in mice. Male mice are infertile, whereas female mice show age-related infertility, reminiscent of primary ovarian insufficiency. The reduced efficiency in synapsis in fetal oocytes may result in a lower ovarian reserve to be established [50] In human males, variants in RAD21L have been implicated in meiotic arrest and Sertoli cell-only syndrome [53].

Variants in seven genes coding for components of the SC were also investigated in this candidate gene group. Of the genes coding for components of the central element of the SC (i.e., *SYCE1*, *SYCE2*, *SYCE3*, *TEX12*), *SYCE2* showed a statistically significant association (in the MI mothers vs. fathers) (Fig 2), although the association with *SYCE3* was close to the cutoff for significance (also in MI mothers vs. fathers).

The other SC genes we examined code for the transverse filament (*SYCP1*) and components of the axial/lateral elements (*SYCP2* and *SYCP3*). *SYCP1* showed significant association in the MI vs. MII analysis, but not in the other analyses (Fig 3). The signal in *SYCP1* was primarily located at an imputed SNP, at rs35401563, so this result requires confirmation by further genotyping. *SYCP2* showed highly significant associations in both the MII mothers vs. fathers and the MI vs. MII comparisons (Fig 4), suggesting the potential for an effect specific to MII. *SYCP3* was nearly significant in the MI vs. MII comparison.

Among the other candidate genes in this group, the only statistically significant result was for *MND1*. The observed significant association was strongest in the MI vs. MII comparison, was also strong in the MII vs. fathers comparison, and was much weaker in the MI vs. fathers comparison. This pattern suggests that this locus may be associated with MII nondisjunction. Based on genetic and cellular analysis of deletion mutants, MND1, acting with HOP2, plays a role in the initial processing of DSBs. Specifically, the HOP2-MND1 complex is involved in two separate stages of the DMC1-promoted recombination process: first, in the stabilization of DMC1 filaments on the resected end of the DSBs, and second, in the promotion of the subsequent strand invasion steps. In higher eukaryotes (mouse [54] and *Arabidopsis thaliana* [55, 56]), MND1 appears to be required for normal male and female fertility. Mutations result in normal recombination initiation, but meiotic DSBs are abnormally repaired and chromosome synapsis is aberrant [17]. The HOP2-MND1 complex has also been implicated in ovarian dysfunction and biochemically, is capable of driving RAD51-mediated alternative lengthening of telomeres in somatic cells [57]. If this association is confirmed, understanding why the effect of the variant is stronger in MII errors vs. MI errors may shed more light on its function.

#### Genes associated with human genome-wide recombination counts (shown in the bottom half of Table 2, below the double line of Table 2)

We also examined the eight regions identified in Kong et al. that were highly associated with genome-wide recombination counts in a large Icelandic study of 71,929 parent-offspring pairs [33]. Of the eight regions, three showed associations with maternal nondisjunction that were statistically significant according to the cutoffs shown in Table 2. The first region included *RAD21L* for which results are discussed above, as it was also in the group of candidate genes for meiotic processes. The second statistical signal was in the region of *SMEK1* (also known as protein phosphatase 4 regulatory subunit 3 (*PPP4R3A*)) and was strongest in the MI vs. MII mothers comparison (Fig 5). SMEK1 is known as a regulator of cellular functions, including apoptosis, cell growth, microtubule organization, cell cycle arrest, and TNF and PI3K/Akt signaling (e.g., [58, 59]). It is also known to play a role in endothelial cell function and subsequent angiogenesis [60]. However, its role in meiosis is unknown, although it is known to be expressed in the ovary.

The third signal is in the region of CCDC43, and was evident in both the MI mothers vs. fathers analysis and the MII mothers vs. fathers analysis (see S7 Fig). There is no known function of CCDC43 in meiosis. In the study of Kong et al. [33], the SNP associated with recombination (rs75502650) was located in an intron of CCDC43. It was estimated to increase the global recombination rate by 76 cM and this effect was limited to females.

### Strongest results from the genome-wide association study

Because of the limited sample size in this study, the full GWAS produced only suggestive results, though a few of those top results have strong support in the literature for the relevance of the gene functions to meiosis or fetal survival. Manhattan plots and Q-Q plots for each GWAS analysis are included in the Supporting Information (S10 Fig). Tables 3–7 show the most statistically significant results from each of the comparisons in the genome-wide association study. For each result for a given comparison, the corresponding table also gives the smallest p-value within 20kb in each of the other comparisons. Detailed results are included in the Supporting Information (S3–S7 Tables).

**Table 3:** Top hits from the all mothers vs. fathers genome-wide association study.

Locus	All mothers vs. fathers	MI mothers vs. fathers	MII mothers vs. fathers	MI mothers vs. MII mothers	TDT
*rs10948101*	P = 7.65e-07	P = 1.58e-06	P = 0.000897	P = 0.0634	P = 0.00514
*rs35141718*	P = 2.02e-06	P = 1.92e-05	P = 0.0109	P = 0.127	P = 0.0499
*rs11535058*	P = 2.42e-06	P = 2.1e-05	P = 7.69e-05	P = 0.0411	P = 0.015
*rs62086686*	P = 4.45e-06	P = 0.000292	P = 8.53e-05	P = 0.0171	P = 0.0133
*rs75733466*	P = 5.09e-06	P = 0.00023	P = 1.24e-05	P = 0.00189	P = 0.0212
*rs117746305*	P = 5.14e-06	P = 0.000168	P = 3.93e-06	P = 0.056	P = 0.0881
*rs12947774*	P = 5.62e-06	P = 0.000199	P = 0.000949	P = 0.00229	P = 0.0339
*rs1612273*	P = 5.93e-06	P = 7.22e-05	P = 1.62e-05	P = 0.000934	P = 0.00432
*rs12652455*	P = 6.43e-06	P = 2.43e-05	P = 0.000437	P = 0.0429	P = 0.0518
*rs148846406*	P = 6.79e-06	P = 3.85e-05	P = 0.00673	P = 0.0562	P = 0.00883
*rs11026040*	P = 8.22e-06	P = 0.000238	P = 3.1e-05	P = 0.00505	P = 0.00794
*rs7010571*	P = 8.27e-06	P = 3.41e-05	P = 0.0185	P = 0.0796	P = 0.0477
*rs35816728*	P = 9.96e-06	P = 6.09e-05	P = 0.00101	P = 0.00124	P = 0.00815

Suggestive associations (p < 10^−5^) are recorded (highlighted cells). For each such locus, the most significant association within 20kb is recorded for each of the other four genome-wide analyses. Rows are ordered by significance. (MI: meiosis I; MII: meiosis II; P: p-value.)

**Table 4:** Top hits from the MI mothers vs. fathers genome-wide association study.

Locus	All mothers vs. fathers	MI mothers vs. fathers	MII mothers vs. fathers	MI mothers vs. MII mothers	TDT
*rs10948100*	P = 7.65e-07	P = 1.58e-06	P = 0.000897	P = 0.0634	P = 0.00514
*rs35288347*	P = 1.26e-05	P = 2.72e-06	P = 0.023	P = 0.0298	P = 0.0339
*rs4649043*	P = 0.000148	P = 3.1e-06	P = 0.00388	P = 0.000408	P = 0.00556
*rs437933*	P = 1.79e-05	P = 3.29e-06	P = 0.0683	P = 0.05	P = 0.0186
*rs16847735*	P = 2.44e-05	P = 3.77e-06	P = 0.0173	P = 0.0135	P = 0.0617
*rs2467011*	P = 5.02e-05	P = 4.29e-06	P = 0.0902	P = 0.00786	P = 0.158
*rs9442389*	P = 1.88e-05	P = 5.27e-06	P = 0.0701	P = 0.0164	P = 0.0241
*rs731245*	P = 0.000148	P = 6.91e-06	P = 0.00772	P = 0.000809	P = 0.00654
*rs984968*	P = 1.48e-05	P = 6.95e-06	P = 0.0741	P = 0.0425	P = 0.0833
*rs9984132*	P = 5.86e-05	P = 9.87e-06	P = 0.0539	P = 0.00662	NA

Suggestive associations (p < 10^−5^) are recorded (highlighted cells). For each such locus, the most significant association within 20kb is recorded for each of the other four genome-wide analyses. Rows are ordered by significance. (MI: meiosis I; MII: meiosis II; P: p-value.)

**Table 5:** Top hits from the MII mothers vs. fathers genome-wide association study.

Locus	All mothers vs. fathers	MI mothers vs. fathers	MII mothers vs. fathers	MI mothers vs. MII mothers	TDT
*rs1855111*	P = 6.48e-05	P = 0.00213	P = 2.2e-06	P = 0.00698	P = 0.00494
*rs76740710*	P = 7.19e-06	P = 0.000236	P = 3.93e-06	P = 0.0341	P = 0.00648
*rs12981234*	P = 0.0013	P = 0.00911	P = 4.28e-06	P = 8.8e-05	P = 0.0854
*rs200216460*	P = 0.000209	P = 0.0104	P = 4.99e-06	P = 0.00373	P = 0.00284
*rs11668205*	P = 0.00588	P = 0.00218	P = 5.49e-06	P = 3.34e-05	P = 0.0233
*rs146838878*	P = 0.0197	P = 0.0249	P = 6.16e-06	P = 0.000328	P = 0.000463
*rs115281615*	P = 0.047	P = 0.0792	P = 6.34e-06	P = 7.33e-07	P = 0.0712
*rs62359711*	P = 0.0024	P = 0.00108	P = 6.91e-06	P = 0.000191	P = 0.0112
*rs73178888*	P = 0.00754	P = 0.0041	P = 7.05e-06	P = 1.72e-05	P = 0.0122
*rs9966603*	P = 0.00967	P = 0.0639	P = 7.05e-06	P = 0.00208	P = 0.00759
*rs13020106*	P = 0.00149	P = 0.0335	P = 7.12e-06	P = 0.00336	P = 0.0162
*rs2560850*	P = 0.0016	P = 0.00365	P = 7.69e-06	P = 0.000332	P = 0.00604
*rs1191234*	P = 0.00176	P = 0.0137	P = 9.98e-06	P = 0.00157	P = 0.0411

Suggestive associations (p < 10^−5^) are recorded (highlighted cells). For each such locus, the most significant association within 20kb is recorded for each of the other four genome-wide analyses. Rows are ordered by significance. (MI: meiosis I; MII: meiosis II; P: p-value.)

**Table 6:** Top hits from the MI mothers vs. MII mothers genome-wide association study.

Locus	All mothers vs. fathers	MI mothers vs. fathers	MII mothers vs. fathers	MI mothers vs. MII mothers	TDT
*rs115281615*	P = 0.047	P = 0.0792	P = 6.34e-06	P = 7.33e-07	P = 0.0712
*rs6440985*	P = 0.0958	P = 0.0112	P = 0.000198	P = 1.31e-06	P = 0.00796
*rs2806747*	P = 0.0934	P = 0.0656	P = 0.00037	P = 1.35e-06	P = 0.00284
*rs9319652*	P = 0.0225	P = 0.0267	P = 0.00243	P = 4.09e-06	P = 0.039
*rs11977478*	P = 0.00474	P = 0.00902	P = 0.00019	P = 4.18e-06	P = 0.00232
*rs11034351*	P = 0.0124	P = 0.00284	P = 7e-04	P = 4.3e-06	P = 0.0295
*rs7685548*	P = 0.00618	P = 0.000955	P = 0.00884	P = 4.82e-06	P = 0.00226
*rs71967233*	P = 0.044	P = 0.0785	P = 0.000135	P = 5.24e-06	P = 0.0163
*rs34282937*	P = 0.0334	P = 0.0368	P = 5.95e-05	P = 5.48e-06	P = 0.0298
*rs77525287*	P = 0.0707	P = 0.000444	P = 0.0124	P = 5.9e-06	P = 0.099
*rs4818884*	P = 0.0454	P = 0.0312	P = 0.00354	P = 9.57e-06	NA
*rs61999085*	P = 0.00851	P = 0.000125	P = 0.0449	P = 9.84e-06	P = 0.00243

Suggestive associations (p < 10^−5^) are recorded (highlighted cells). For each such locus, the most significant association within 20kb is recorded for each of the other four genome-wide analyses. Rows are ordered by significance. (MI: meiosis I; MII: meiosis II; P: p-value.)

**Table 7:** Top hits from the TDT (transmission disequilibrium test) genome-wide association study.

Locus	All mothers vs. fathers	MI mothers vs. fathers	MII mothers vs. fathers	MI mothers vs. MII mothers	TDT
*rs3802065*	P = 0.0144	P = 0.0066	P = 0.0318	P = 0.00859	P = 4.84e-07
*rs2867076*	P = 0.0164	P = 0.0227	P = 0.0081	P = 0.00904	P = 1.05e-06
*rs7451700*	P = 0.0186	P = 0.0169	P = 0.0544	P = 0.0195	P = 1.71e-06
*rs7389783*	P = 0.0421	P = 0.0397	P = 0.00488	P = 0.00741	P = 1.93e-06
*rs17769147*	P = 0.182	P = 0.152	P = 0.0738	P = 0.0624	P = 2.06e-06
*rs201634098*	P = 0.00592	P = 0.00385	P = 0.0722	P = 0.015	P = 3.52e-06
*chr23:154539980*	NA	NA	NA	NA	P = 3.63e-06
*rs74615884*	P = 0.0801	P = 0.037	P = 0.0167	P = 0.00236	P = 3.73e-06
*rs158866*	P = 0.036	P = 0.00582	P = 0.0103	P = 0.0102	P = 3.77e-06
*rs1187600*	P = 0.0553	P = 0.0636	P = 0.204	P = 0.156	P = 6.25e-06
*rs55743346*	P = 0.0514	P = 0.0371	P = 0.0834	P = 0.0234	P = 7.34e-06
*rs183199067*	P = 0.0131	P = 0.00285	P = 0.0396	P = 0.0254	P = 9.55e-06
*rs140022090*	P = 0.0163	P = 0.00733	P = 0.0538	P = 0.00493	P = 9.55e-06
*rs34518363*	P = 0.00538	P = 0.00186	P = 0.049	P = 0.0394	P = 9.55e-06
*rs6681167*	P = 0.0358	P = 0.0505	P = 0.0648	P = 0.0377	P = 9.58e-06

Suggestive associations (p < 10^−5^) are recorded (highlighted cells). For each such locus, the most significant association within 20kb is recorded for each of the other four genome-wide analyses. Rows are ordered by significance. (MI: meiosis I; MII: meiosis II; P: p-value.)

#### rs10948101 on chromosome 6 near *VEGFA*

**Fig 6:**
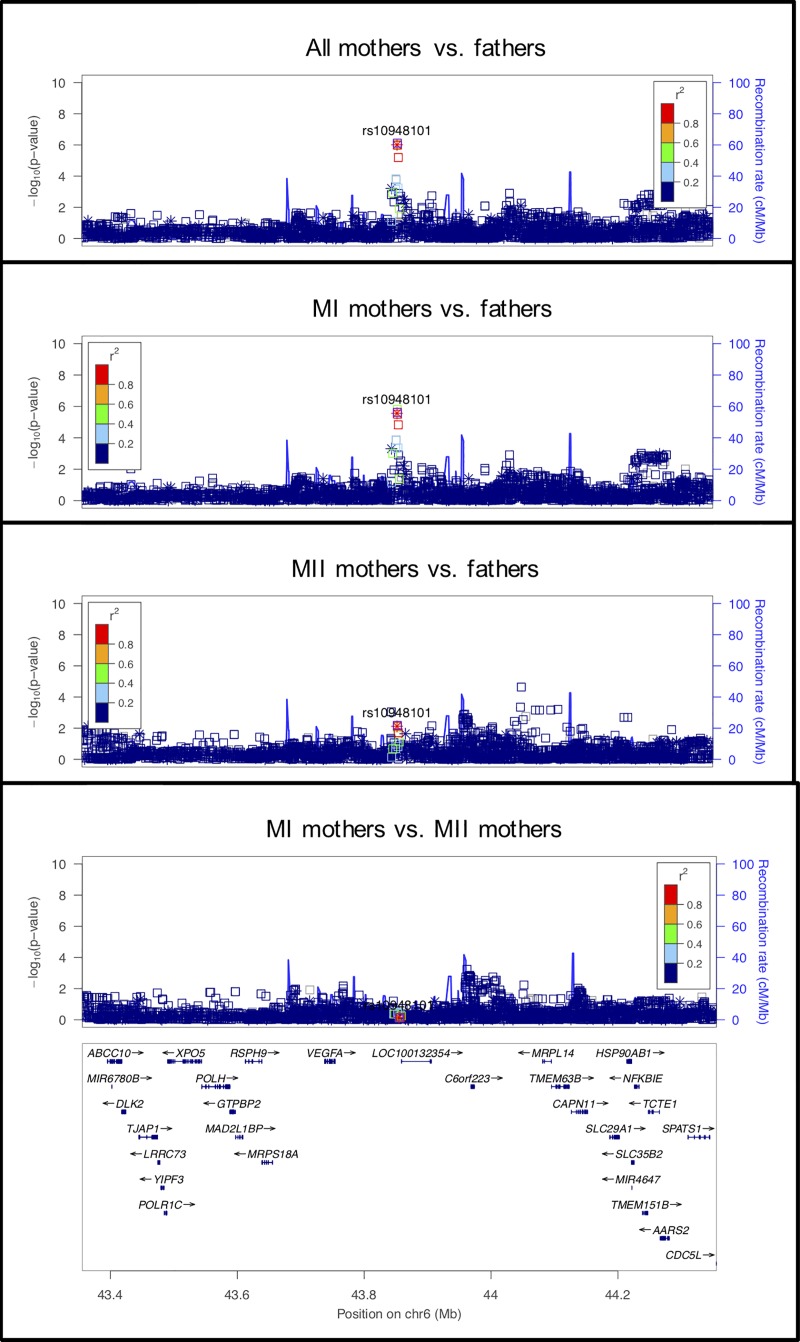
LocusZoom plot for *VEGFA*.

The observed signal for this locus was strongest for the analysis of all mothers vs. fathers and was located within *LOC100132354*, a long non-coding RNA (lncRNA) (Fig 6). Upstream of this intergenic lncRNA is *VEGFA*, the gene encoding vascular endothelial growth factor A (VEGFA). In a recent meta-analysis, *LOC100132354* was confirmed to be highly associated with VEGF circulating levels in serum [61]. VEGFA plays multiple roles in ovarian development and function (reviewed in McFee and Cupp [62]). Vascularization plays a role in the formation of early ovarian structures, primordial follicle assembly, and follicle activation. Further, ovarian function is highly dependent on the development and continual remodelling of a complex vascular system. This allows the follicle and the corpus luteum to receive the needed oxygen, nutrients, and systemic hormones and the release of ovarian hormones (reviewed in Robinson et al. [63]). If angiogenesis is disrupted, follicular growth is reduced, ovulation is perturbed, and development and function of the corpus luteum is significantly altered. The action of VEGFA is necessary at all these stages of development. We did not see any significant effect in the TDT analysis, so there is no suggestion that this locus is associated with survival to term.

#### rs11535058 on chromosome 2 near *SLC39A10*

**Fig 7:**
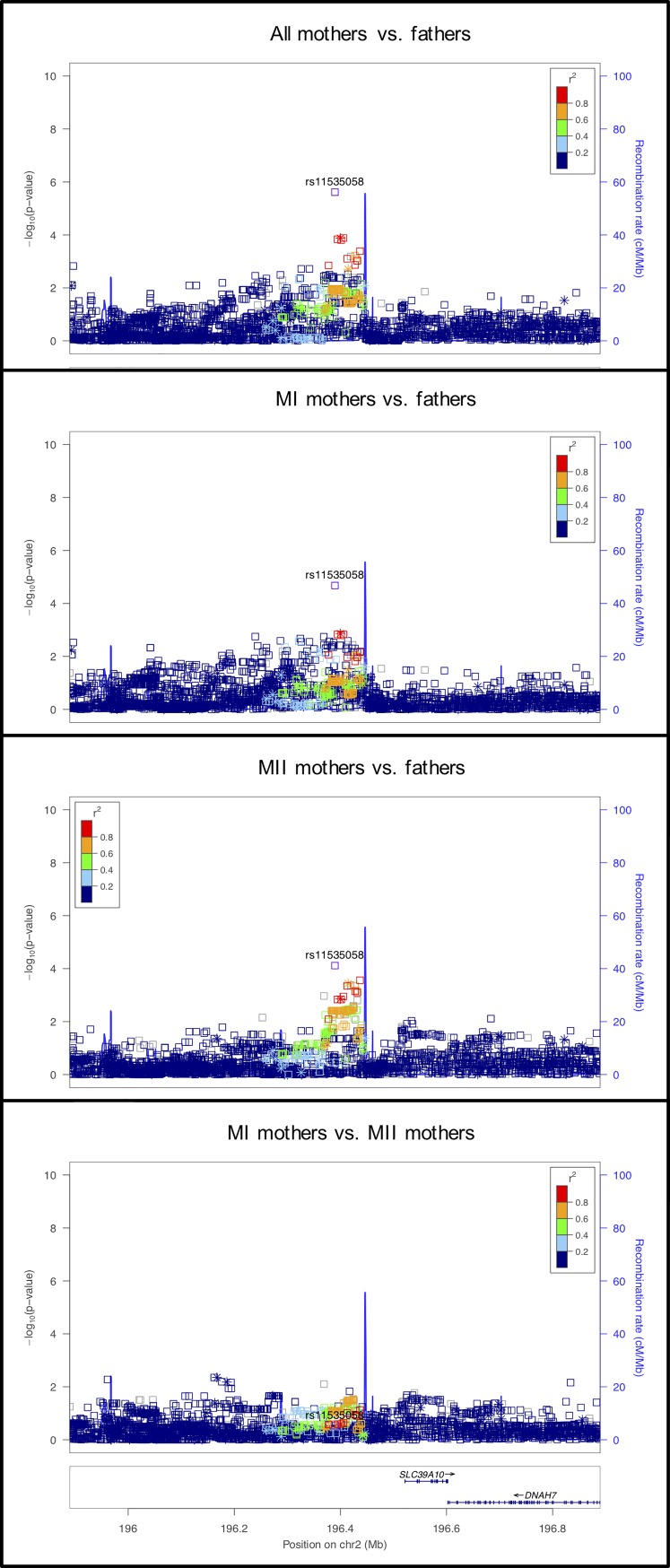
LocusZoom plot for *SLC39A10*.

The observed signal in this region, strongest in the mothers vs. fathers comparison, was located 132kb downstream of *SLC39A10* (Fig 7), with a similar, but non-significant, effect size for both MI mothers vs. fathers and MII mothers vs. fathers. *SLC39A10* is involved in the zinc transport network. Regulation of intracellular zinc is essential for oocyte maturation and activation. In mouse, progression of the oocyte from a cell arrested in prophase of MI into a mature egg arrested at metaphase of MII is accompanied by an increase in total zinc content. This increase is required for proper meiotic progression [64]. Also, exit from MII during oocyte activation requires decreasing cellular zinc through the rapid export of zinc from the oocyte. These ‘zinc sparks’ are required for oocyte activation and resumption of the cell cycle [65].

Specific to *SLC39A10*, Lisle et al. [66] found a complex zinc transport network present in the cumulus-oocyte complex in mouse oocytes. They found that mRNA transcripts for specific zinc transporter proteins (SLC family), including Slc39a10 were higher in oocytes, while another unique set of zinc transporter protein transcripts were higher in cumulus cells. Thus, zinc homeostasis, regulated in the cumulus-oocyte complex, may affect both MI and MII processes.

#### rs35288347 on chromosome 19 near *AURKC*

**Fig 8:**
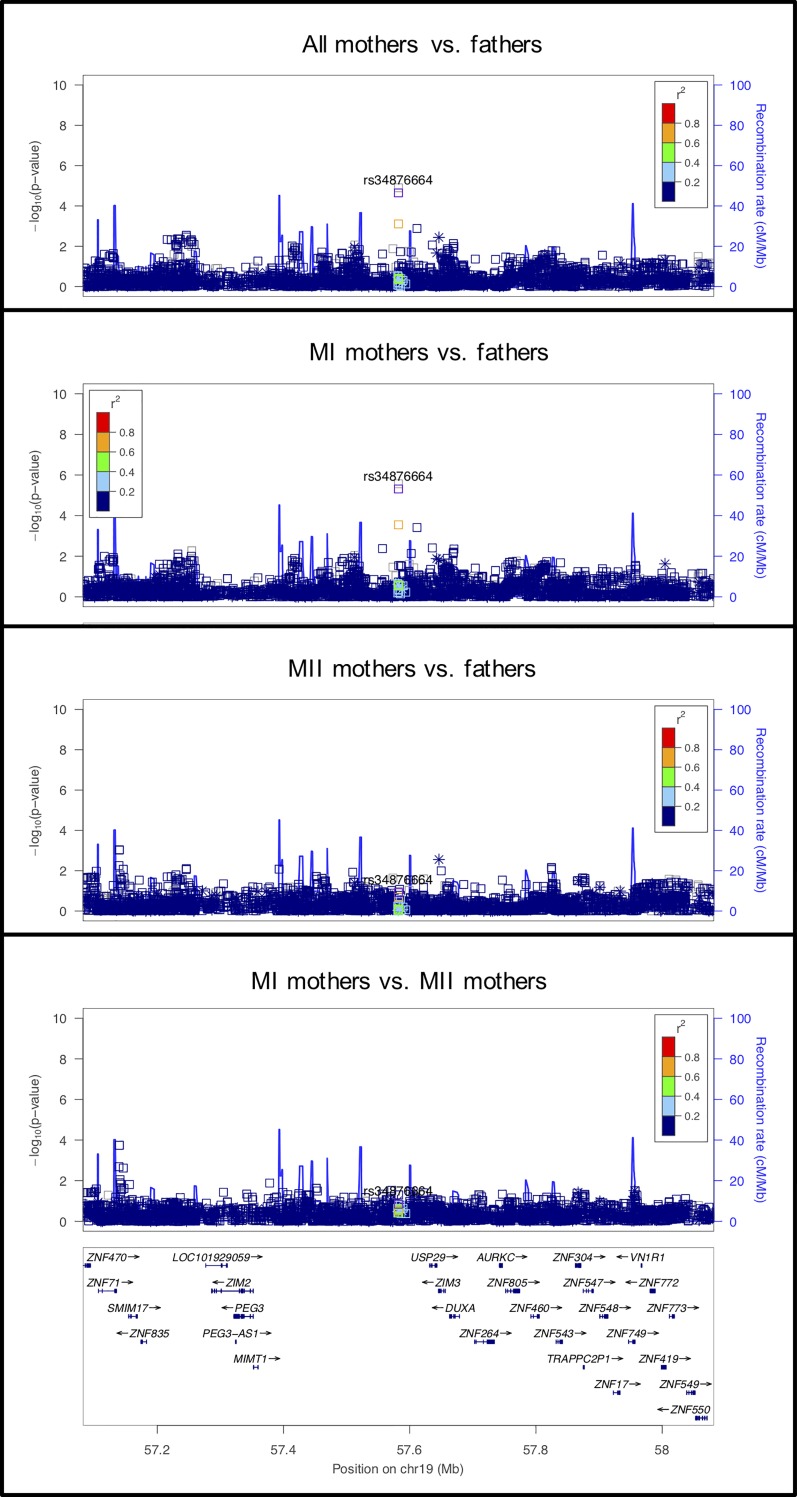
LocusZoom plot for *AURKC*.

This signal occurs primarily in the MI mothers vs. fathers analysis (Fig 8), with a similar but less significant effect in the mothers vs. fathers analysis. Consistent with the possibility of an effect specific to MI, mutations in the *AURKC* gene in this region (160kb away) cause tetraploidy in human sperm and MI arrest in mouse oocytes [67, 68].

#### rs9984132 on chromosome 21 located in a gene rich region

**Fig 9:**
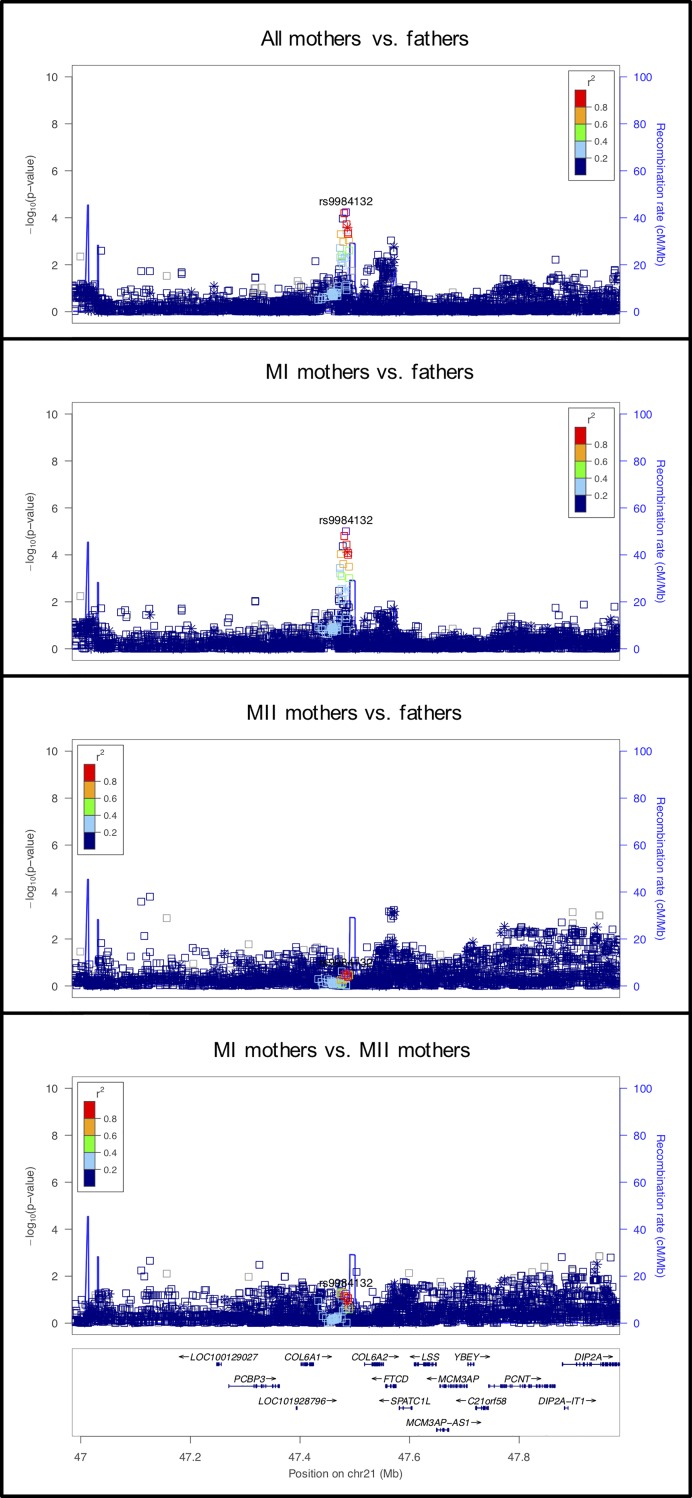
LocusZoom plot for *rs9984132* on chromosome 21.

The signal at rs9984132 on chromosome 21 is located in a gene-rich region (Fig 9). The strongest signal at this locus was identified in the comparison of MI mothers with fathers. Two genes stand out as possible candidates for involvement in chromosome segregation. *COL6A2*, located 34kb upstream of the signal, codes for one of the components of collagen that is part of the extracellular matrix (ECM) formed by cumulus cells. This ovarian follicular ECM is related to proliferation, steroidogenesis, and luteinization [69]. As the formation of this ECM is involved in fertilization and embryo quality, the observation that this signal appears to be only related to MI nondisjunction reduces the support of *COL6A2* as a candidate.

*PCNT* is a gene located about 260kb upstream from the signal. Pericentrin, coded by *PCNT*, is a highly conserved component of the acentriolar microtubule-organizing centers (aMTOCs) in mouse oocytes. aMTOCs play a vital role in meiotic spindle assembly and stability. Depletion of pericentrin in mouse oocytes leads to increased rates of aneuploidy [70]. Human oocytes differ from mouse oocytes in that they lack *PNCT* and aMTOCs in MI, where spindle assembly is mediated from chromosomes by the small guanosine triphosphates [71]. Thus, more work is needed to confirm this signal and its underlying genetic association.

#### rs73178888 on chromosome 8 near a region associated with meiotic recombination

**Fig 10:**
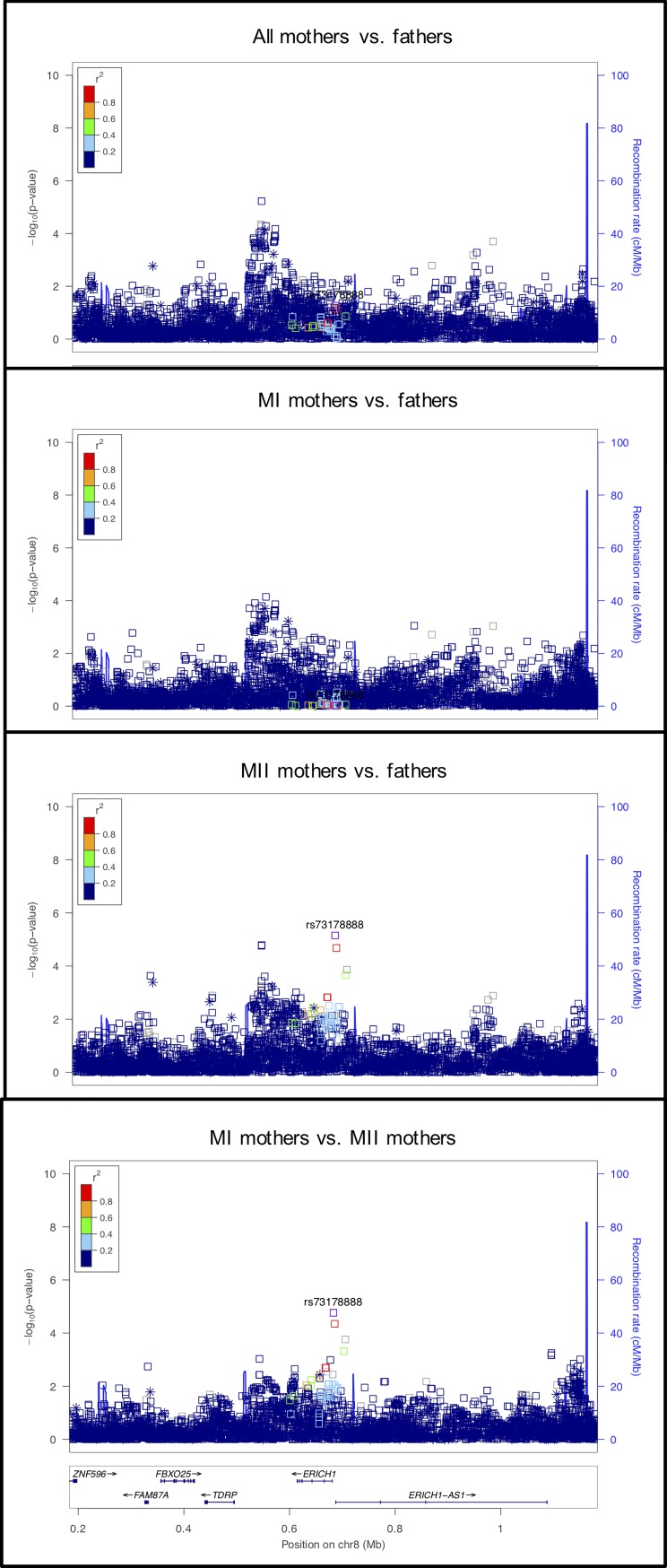
LocusZoom plot for *DLGAP2*.

This signal, located in an intron of *ERICH1*, is primarily observed in the MII mothers vs. fathers analysis and in the MI vs. MII analysis, suggesting that it might be an MII risk locus (Fig 10). The location is noteworthy because Begum et al. [72] identified a variant 59kb away in this region as potentially associated with meiotic recombination (specifically recombination outside of hotspots) in a euploid population. There is no evidence to suggest that *ERICH1* or *DLGAP2* (also known as *ERICH1-AS1)*, 3kb from the signal, is involved in recombination or meiosis. The next closest gene is *TDRP*. The deficiency of *TDRP* in mice is suggested to be involved in sperm motility and may play a role in spermatogenesis [73], but there is no evidence for involvement is oogenesis.

#### rs115281615 on chromosome 4 near *CPEB2*

**Fig 11:**
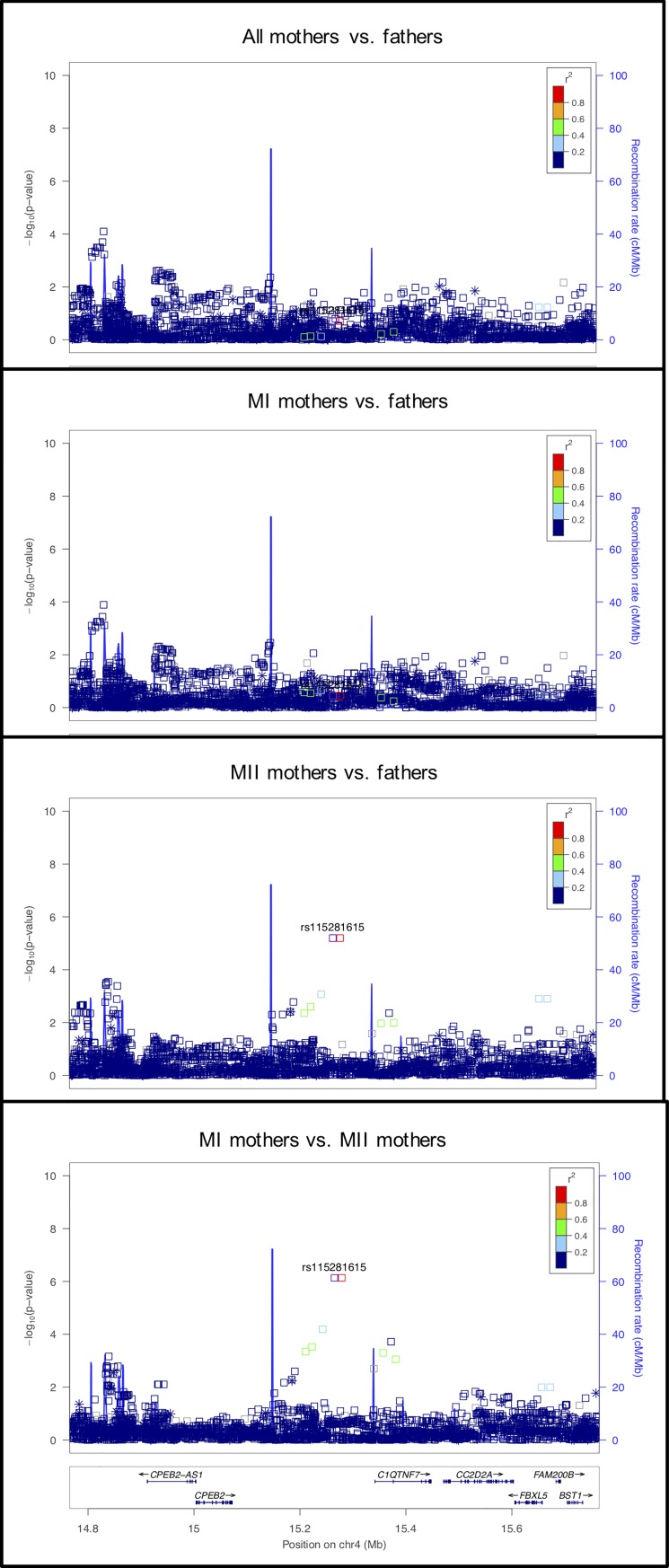
LocusZoom plot for *CPEB2*.

The signal at this locus is primarily observed in the MII mothers vs. fathers comparison (Fig 11). The genes in the region, *C1QTNF7*, *and CC2D2A*, do not have evidence for a role in meiosis. *CPEB2*, located 193kb from the signal, encodes an RNA-binding protein, cytoplasmic polyadenylation element binding protein and is thought to be involved in regulated translation, a system that allows the rapid production of selective proteins in response to a physiological signal. CPEB2 interacts with the elongation factor, eEF2, to slow down peptide elongation of CPEB2-bound RNA [74]. In mice, this protein is highly similar to the family of CPEBs that regulate cytoplasmic polyadenylation of mRNA as a trans-factor in oogenesis and spermatogenesis. CPEB2 is expressed post-meiotically in mouse spermatogenesis, which suggests a possible role in translational regulation of stored mRNAs in transcriptionally inactive haploid spermatids [75].

#### rs2560850 on chromosome 5 in an intron of *MYO10*

**Fig 12:**
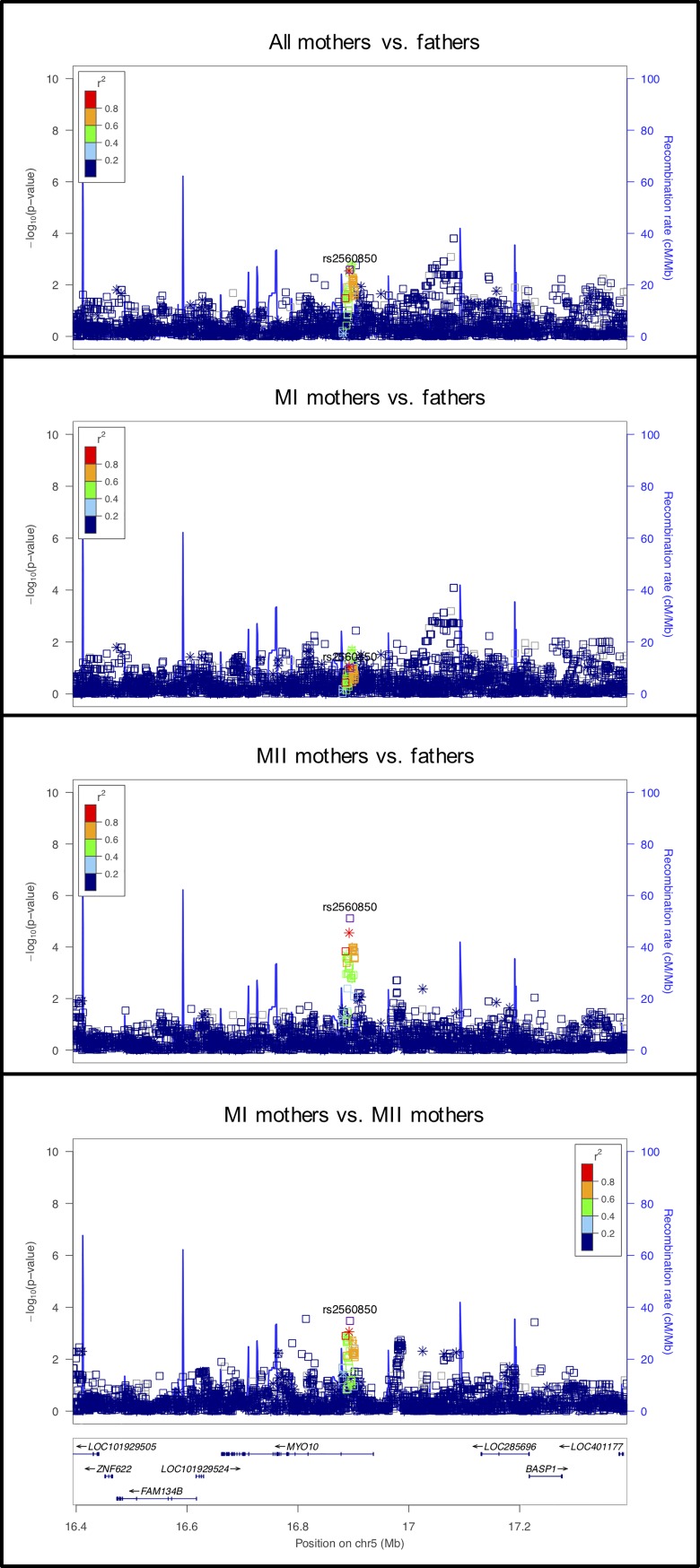
LocusZoom plot for *MYO10*.

The comparison between MII mothers vs. fathers shows the strongest statistical significance for this locus which includes *MYO10* (Fig 12). Myosin-10 proteins are phosphoinositide-binding, actin-based motors that play an important role during meiosis in the integration of the F-actin and microtubule cytoskeletons. Proper spindle positioning and orientation are essential for asymmetric cell division and these functions are particularly important in meiosis. In Xenopus oocytes, Weber et al. [76] showed that myosin-10 is associated with microtubules and is concentrated where the meiotic spindle contacts the F-actin-rich cortex. This observation and others suggest that myosin-10, the microtubule-binding myosin, is required for anchoring the spindle and an actin-binding kinesin is required for meiotic cytokinesis [76, 77]. Recently F-actin was shown to permeate the microtubule spindles in oocytes of many mammals, including human, where it prevents lagging chromosomes and thus segregation errors, including during anaphase I [78].

### Strongest results from the TDT analyses

Together with the other case/control association tests to identify genes associated with nondisjunction, we also had the opportunity to use the TDT. We assumed that the TDT might help tease apart genes associated with nondisjunction from those associated with fetal “survival genes.” If there is association between *maternal* genotype and either nondisjunction or survival, this test can theoretically identify it. We only performed this test in our GWAS approach, not our candidate genes, since the candidates were chosen for possible involvement in nondisjunction per se. Here we highlight two genes where the statistical signal was relatively strong, although not genome-wide significant.

The first is located at rs17769147, 24kb upstream of the gene *RNF182*, which encodes a RING-finger-containing transmembrane protein that includes an E3 ubiquitin ligase activity. There was no statistical signal for any of the case/control comparisons, only for the TDT. Studies have shown that this gene is expressed preferentially in the brain and is up-regulated in Alzheimer’s disease brain and in neuronal cell cultures that are subject to stress-induced cell death [79]. In another line of study, *RNF182* was found to be one of the gene targets of MeCP2, the gene involved in Rett syndrome. The group of identified gene targets are involved in the regulation of the cell growth and survival of neuronal cells [80].

Another strong TDT signal was found at rs158866, with no statistical signal for any of the case/control comparisons. This signal is located within *NEDD4L* (also referred to as *NEDD4-2*), a gene encoding a ubiquitin ligase. This protein binds and regulates membrane-associated proteins (although not exclusively), particularly ion channels and transporters (reviewed in Goel et al. [81]). NEDD4L interacts with several other proteins and may regulate other important substrates as well (e.g., [82, 83]). Based on current evidence, this ligase is essential for the maintenance of cellular homeostasis.

## Discussion

We present, for the first time, a candidate gene study and GWAS of chromosome 21 maternal nondisjunction. The goal of this project was to gain insight into factors that may predispose a woman to this common chromosomal error.

### Genes associated with cohesin complex

The meiosis-specific cohesin subunits are encoded by *SMC1β*, *REC8*, *RAD21L*, and *STAG3*. Of these genes, we found that variation in *RAD21L* was associated with nondisjunction, with the strongest signal with MI nondisjunction. As part of the cohesion complex [47–50], RAD21L plays a role in the structural maintenance of chromosomes (SMC) complex. The SMC complex includes cohesin, condensin and SMC5/6, and is an important regulator of chromosome dynamics and structure during both mitosis and meiosis. In female mice, mutations in meiosis-specific cohesins and in the SMC complex increase the frequency of oocyte aneuploidy and primary ovarian insufficiency [50, 84–86]. The study of Kong et al. [33] found a highly significant association of *RAD21L* with male recombination and a much weaker signal in females; Begum et al. [72] did not replicate this finding, although the genomic region had poor coverage in two of the three datasets of their study. Together, this suggests a sex-specific role of RAD21L and one in females that maybe more directly related to segregation of bivalents than recombination counts per se.

There are several possible explanations for why genetic variation in *RAD21L* was associated with maternal nondisjunction whereas variation in *REC8* was not, although both are α-kleisin subunits that are part of the cohesin complex. One possible explanation is that there is reduced power to detect a signal based on allele frequencies of variants in *REC8* compared with *RAD21L*. Alternatively one gene may play a more essential role in meiosis, where variation is not tolerated or not compatible with the oocyte surviving to fertilization. It is known that these genes play unique roles during meiosis and thus have differential effects on the meiotic process [87–89].

### Genes associated with the synaptonemal complex (SC)

The general structure of the SC is highly conserved across yeast and mammals, although the genes and proteins involved are not always conserved (reviewed in Cahoon and Hawley [10]). The tripartite protein structure extends along the entire length of the synapsed homologues and assembles alongside cohesin and cohesin-like proteins that hold the sister chromatids of the homologues together (e.g., [90, 91]). Mutations in genes coding for SC components have been identified previously among women with infertility or recurrent miscarriages (reviewed in Geisinger and Benavente [92]). At this time, only mutations in *SYCP3* and *SYCE1* have been identified, but most studies had <100 women available for study. In our study, we found evidence for an association of variants in genes of all three SC components with maternal nondisjunction. We had supporting evidence for *SYCP3* (although not statistically significant), with the strongest statistical signal being found in the comparison of MI vs. MII. In addition, we found a statistically significant association of *SYCE2* in MI vs. fathers and with *SYCP2* in the comparison of MI vs. MII. Thus, with an increased sample size and a more homogeneous reproductive outcome, we were able to confirm the importance of the SC structure for proper segregation of chromosome during human oogenesis.

### Association with recombination-related variants

It is now well established that there is both significant sex-specific and individual variation in genome-wide recombination counts and location of events, in spite of the need for the recombination process to be tightly controlled [93–95]. When there are alterations in the number of recombinants (reduced or no recombination) or their location (pericentromeric or telomeric), there is a high risk for human chromosome nondisjunction [96–98]. Variation in genes that play a role in recombination has been identified and we examined those that were identified in a large Icelandic study using linkage analyses of live births [33]. Begum et al. [72] also attempted to replicate the association of these variants in a population of primarily Northern European ancestry. Their GWAS meta-analyses were extended to the study of recombination phenotypes, including the average recombination count along with those related to placement relative to historical recombination hotspots. Here, we asked whether these variants would also explain susceptibility to maternal nondisjunction. Our results are interesting both with respect the identification of associated regions and to the lack of evidence in the others.

Both Kong et al. [33] and Begum et al. [72] found a strong association of *SMEK1* (also known as *PPP4R3A*) with recombination in females only. Our results identified the strongest association in the comparison of MI vs. MII nondisjunction, which suggests a stage-specific role of this protein, as well as a sex-specific role identified in the recombination studies. At this time, there is no known role of *SMEK1* in meiosis. It is a member of the PP2A subfamily. PP2A is involved in de-protection of centromere cohesin in MII of mammals, a process that is essential for proper sister chromatid segregation (reviewed in Wassmann [99]). Although it is intriguing to think that variation in *SMEK1* may alter this MII-associated process, this is no direct link at this time.

Another locus that deserves follow-up is on chromosome 8 near rs73178888, one of the top hits in our genome-wide analyses. Begum et al. [72] identified this same region as potentially associated with meiotic recombination in a euploid population. Although the genes in the region do not appear to be linked to recombination, further investigation is warranted.

With respect to the two most well-established genes associated with recombination, *RNF212* and *PRDM9*, our data showed no association with nondisjunction. Variation in *RNF212* is sex-specific, some variants being associated with increased recombination in males and others in females [33, 70, 95, 100–102]. RNF212 is known to form many discrete foci along chromosomes early in meiotic prophase I; these foci are then reduced to a few sites where crossovers are formed [103]. For small chromosomes such as chromosome 21, perhaps female-specific variation in this process is less evident compared with genome-wide alterations.

Variation in *PRDM9* is known to be associated with recombination hotspots in both males and females. Kong et al. [33] showed that variants were also associated with total recombination counts in males, but not females. Begum et al. [72] provided further evidence that in females, variants were associated with both recombinant counts within and outside of historical hotspots, in opposite directions. They suggested that females might have a compensatory mechanism, such that increased recombination in hotspots is balanced by decreased recombination elsewhere; thereby not altering the overall recombination count. In males, variants were only associated with recombinants within historical hotspots. In our previous study, we found that historical hotspot usage along maternally-derived nondisjoined chromosomes 21 was similar to that in controls, particularly among MI errors, indicating that the observed altered telomeric placement probably does not involve differential hotspot usage [28]. Subsequently, Oliver et al. [104] studied sequence variation in the zinc finger-binding domain (ZFBD) of *PRDM9* in a subset of the study sample presented here. They found that the frequency of the *PRDM9* ZFBD minor alleles was significantly increased among women who had a chromosome 21 nondisjunction event and no observed recombination on 21q. Further, when these *PRDM9* minor alleles were compared with the major A-allele, fewer predicted binding sites on 21q were found. Together, these observations suggest that allelic variation in *PRDM9* may play a role in nondisjunction, but that the effect size may be small and it may be limited to nondisjunction of achiasmate chromosomes.

### Gene discovery

When we conducted a GWAS, no variants were genome-wide significant; thus, the marginally significant signals need replication prior to additional speculation. We highlighted a few findings in Results for signals in genes that are known to be involved in oogenesis. If these are true signals, our data are consistent with the idea that the underlying susceptibility for chromosome nondisjunction involves different components of oogenesis.

### Conclusion and future direction

Our candidate gene study was successful in detecting statistically significant associations of maternal nondisjunction of chromosome 21 and variation in genes that are essential for proper chromosome segregation during meiosis. Future studies are needed to investigate other known risk factors and their interaction with the genetic variation. For example, stratification by maternal age at the time of birth of the infant with trisomy 21 could provide insight into mechanism of the identified genetic variants. In our exploratory analyses, we did not observe unique age-associated variants; however, our sample sizes were limited. Thus, expansion of the study sample with enrichment of the youngest and oldest maternal age groups would be valuable. Also, studies that further stratify meiotic errors by recombination risk patterns known to increase susceptibility of nondisjunction, namely lack of observed recombination, a single telomeric recombination event or a pericentromeric event, may provide further insight into the function of the genetic variant.

Another possible approach is to examine other sources of samples from which information on aneuploidy may be drawn to obtain larger sample sizes. For example McCoy et al. [105] studied day-3 embryos obtained from *in vitro* fertilization cycles and parents to identify both meiotic and mitotic segregation errors. Irrespective, we have begun to gain insight into which meiotic proteins may be more susceptible to genetic variation, leading to abnormal chromosome segregation. Independent studies are needed to replicate findings from our GWAS study to further identify novel susceptibility genes.

## Supporting information

S1 Fig Manhattan and QQ plot for COHRA study female vs. male analysis. In each Manhattan plot in S1 (and S10), each point is one variant, with the the *x*-axis representing chromosome number and the *y*-axis representing -log_10_(p-value). In each QQ plot, the observed vs. expected quantiles of -log_10_(p-value) are plotted, with the genomic inflation factor lambda shown below.
(TIFF)

S2 Fig LocusZoom plots for candidate loci *BLM*, *DMC1*, *EXOC1*, and *HORMAD1*. In this Figure (and in S3–S9 Figs) the four LocusZoom plots in a row show the results at one locus across all four analyses. Each point is one variant, with the x- and y-axes representing physical position on the chromosome and -log_10_(p-value), respectively. Open squares and asterisks represent genotyped and imputed variants, respectively. Coloring represents linkage disequilibrium (red = stronger, blue = weaker) with the tagging SNP (which is purple). The overlaid blue curve shows the recombination rate.
(TIFF)

S3 Fig LocusZoom plots for candidate loci *HORMAD2*, *MEI1*, *MEI4*, and *MLH1*. (TIFF)

S4 Fig LocusZoom plots for candidate loci *MLH3*, *MND1*, *MSH5*, and *REC8*. (TIFF)

S5 Fig LocusZoom plots for candidate loci *REC114*, *RNF212* (intron), *CCNB1IP1*, and *C14orf39*. (TIFF)

S6 Fig LocusZoom plots for candidate loci *RNF212* (missense variant), *MSH4*, *PRDM9* (upstream variant), and *RNF212* (upstream variant). (TIFF)

S7 Fig LocusZoom plots for candidate loci chr17 inversion, *CPLX1*, *CCDC43*, and *PRDM9* (intergenic variant). (TIFF)

S8 Fig LocusZoom plots for candidate loci *SMC1B*, *SPO11*, *STAG3*, and *SYCE1*. (TIFF)

S9 Fig LocusZoom plots for candidate loci *SYCE3*, *SYCP3*, *TEX12*, and *TRIP13*. (TIFF)

S10 Fig Manhattan and QQ plots for the five genome-wide analyses. (TIFF)

S1 Table Candidate gene results in COHRA study female vs. male analysis. (TIFF)

S2 Table Candidate gene results (detailed). Each row represents one candidate locus (either a gene with a 60kb border on each side or a 60kb window around a SNP). Each column represents an analysis. For each locus-analysis pair, the most significant association at the locus (not always unique) is reported. P-values significant after correcting for multiple testing (i.e., exceeding the Bonferroni-adjusted significance threshold noted in the last column) are marked with an asterisk and highlighted. (MI: meiosis I; MII: meiosis II; P: p-value; OR: odds ratio.) The first 24 loci represent genes selected for their function (above the double line). The latter 13 loci represent SNPs identified by Kong et al. in their GWAS of recombination [33], with annotation in parentheses (below the double line). Note that for each analysis in each gene, S2 Table lists the most statistically significant result within a window, so that the SNP that appears in a given gene is not necessarily the same in each analysis. This also means that some odds ratios appear to “flip” between analyses; for example, a result that shows an odds ratio of 2.0 for one SNP in the MI vs. fathers analysis may be represented as an odds ratio of approximately 0.5 (i.e. 1/2.0) for a nearby SNP in the MII vs. fathers analysis.
(TIFF)

S3 Table Top hits from the all mothers vs. fathers genome-wide association study (detailed). In this table (and in S4–S7 Tables) suggestive associations (p < 10^−5^) are recorded (highlighted cells). For each such locus, the most significant association within 20kb is recorded for each of the other four genome-wide analyses. Rows are ordered by significance. (MI: meiosis I; MII: meiosis II; P: p-value; OR: odds ratio).
(TIFF)

S4 Table Top hits from the MI mothers vs. fathers genome-wide association study (detailed). (TIFF)

S5 Table Top hits from the MII mothers vs. fathers genome-wide association study (detailed). (TIFF)

S6 Table Top hits from the MI mothers vs. MII mothers genome-wide association study (detailed). (TIFF)

S7 Table Top hits from the TDT (transmission disequilibrium test) genome-wide association study (detailed). (TIFF)
