# Supplementary figures and images for: A candidate gene analysis and GWAS for genes associated with maternal nondisjunction of chromosome 21
Source: PLoS Genet. 2019 Dec 12;15(12):e1008414. doi: 10.1371/journal.pgen.1008414 (PMC6932832; doi:10.1371/journal.pgen.1008414)

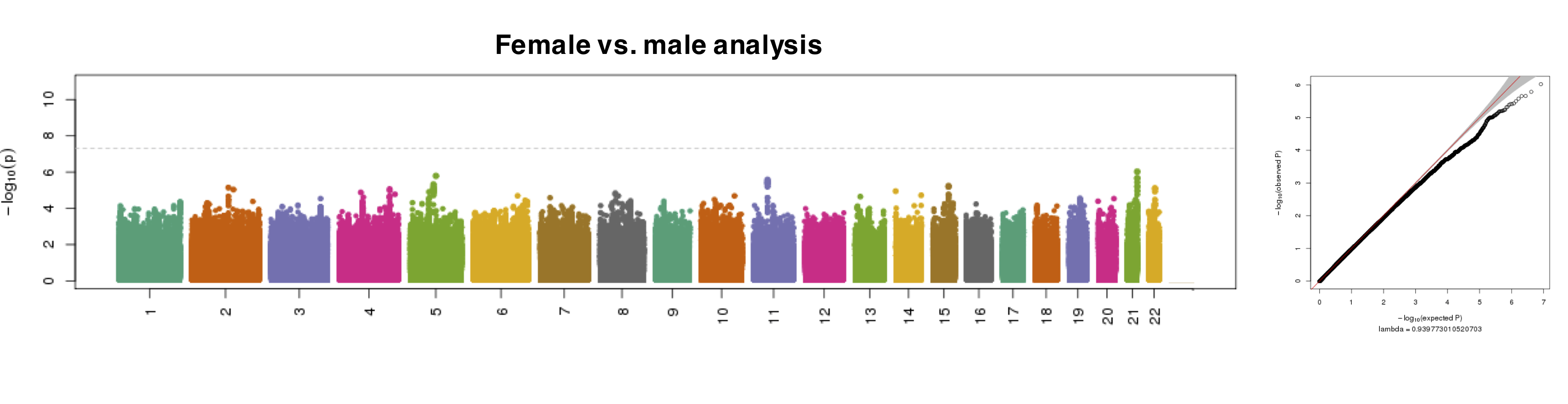

Supplement: S1 Fig — In each Manhattan plot in S1 (and S10), each point is one variant, with the the x-axis representing chromosome number and the y-axis representing -log10(p-value). In each QQ plot, the observed vs. expected quantiles of -log10(p-value) are plotted, with the genomic inflation factor lambda shown below. (TIFF) [file pgen.1008414.s001.tiff]

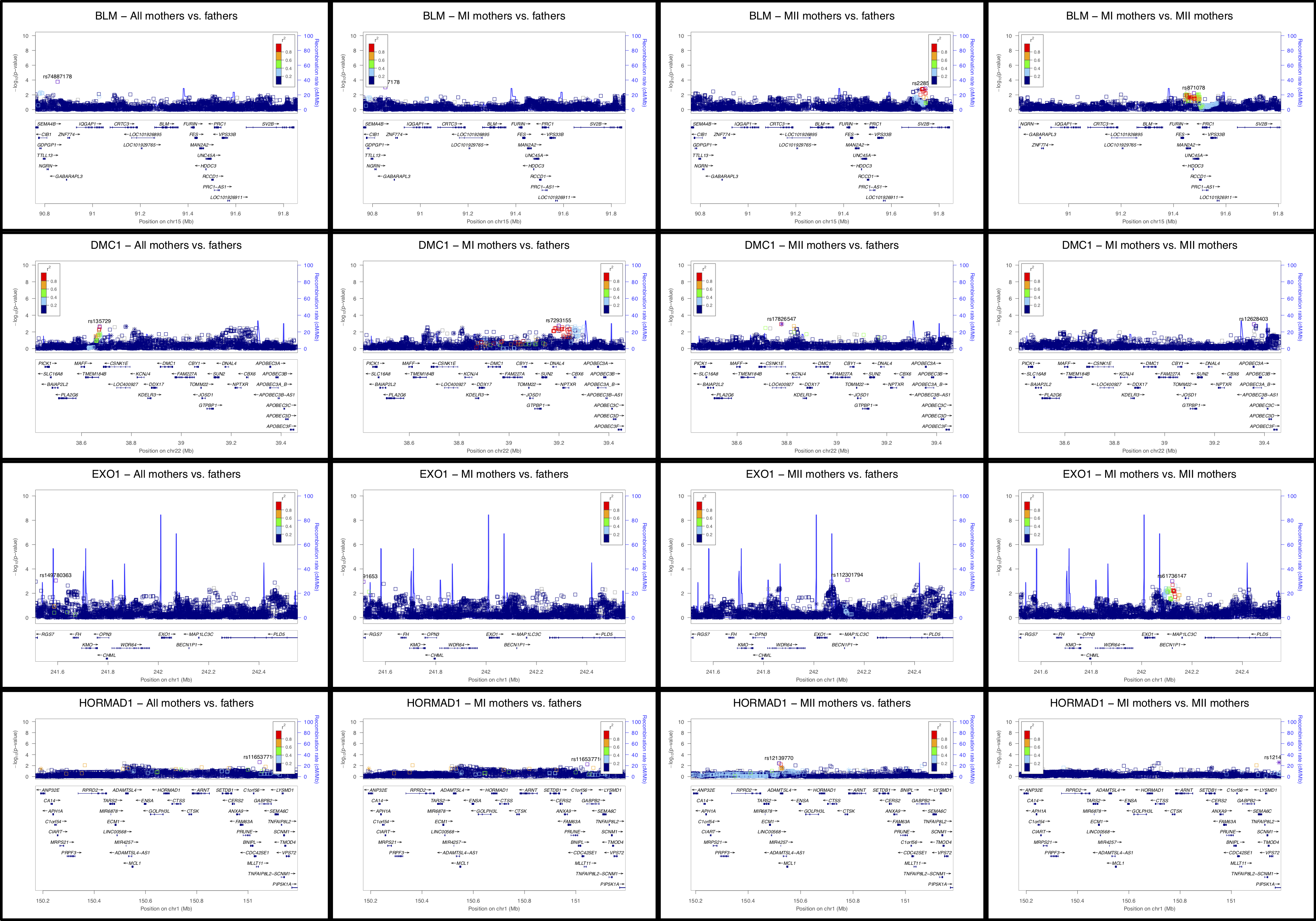

Supplement: S2 Fig — In this Figure (and in S3–S9 Figs) the four LocusZoom plots in a row show the results at one locus across all four analyses. Each point is one variant, with the x- and y-axes representing physical position on the chromosome and -log10(p-value), respectively. Open squares and asterisks represent genotyped and imputed variants, respectively. Coloring represents linkage disequilibrium (red = stronger, blue = weaker) with the tagging SNP (which is purple). The overlaid blue curve shows the recombination rate. (TIFF) [file pgen.1008414.s002.tiff]

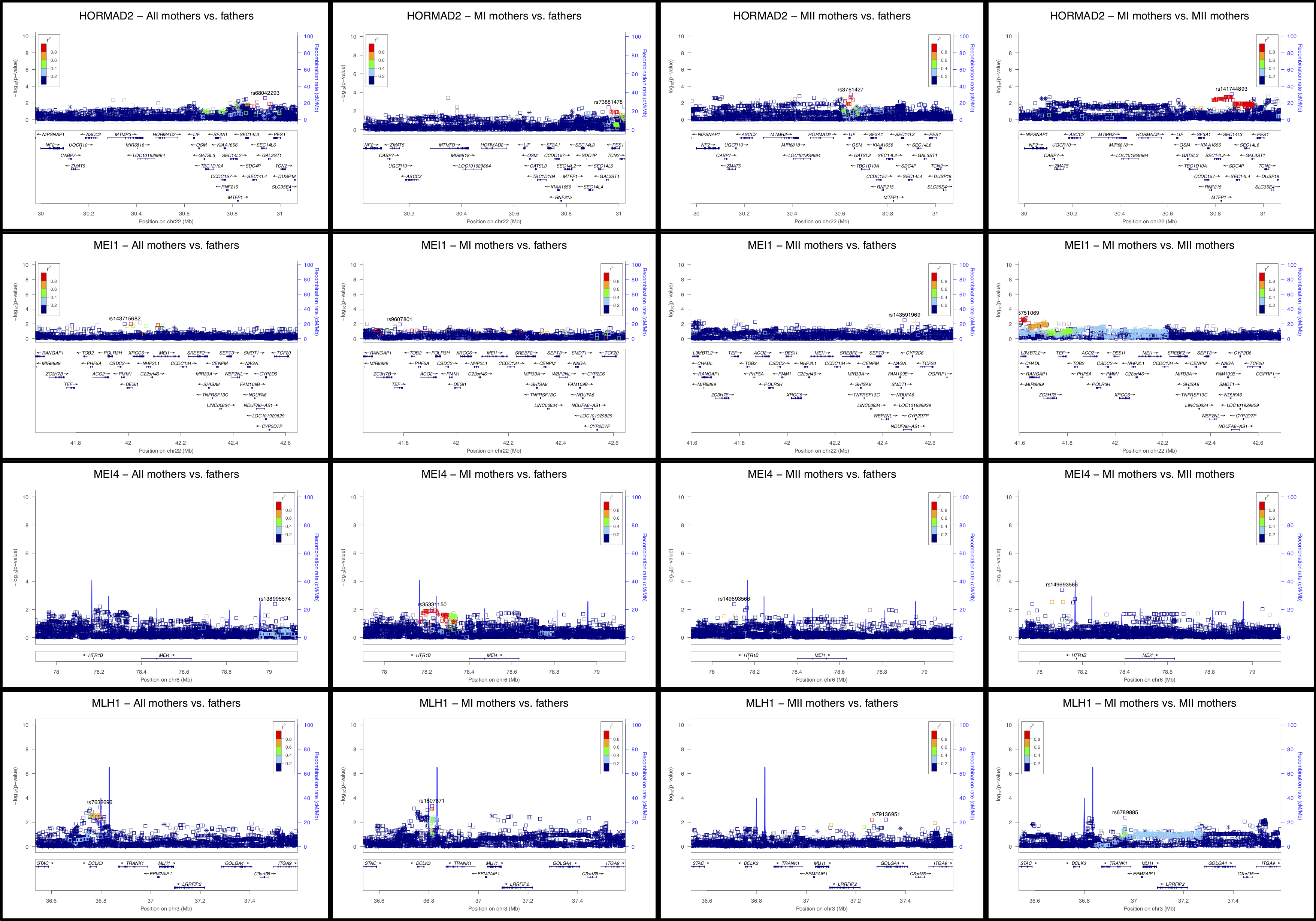

Supplement: S3 Fig — (TIFF) [file pgen.1008414.s003.tiff]

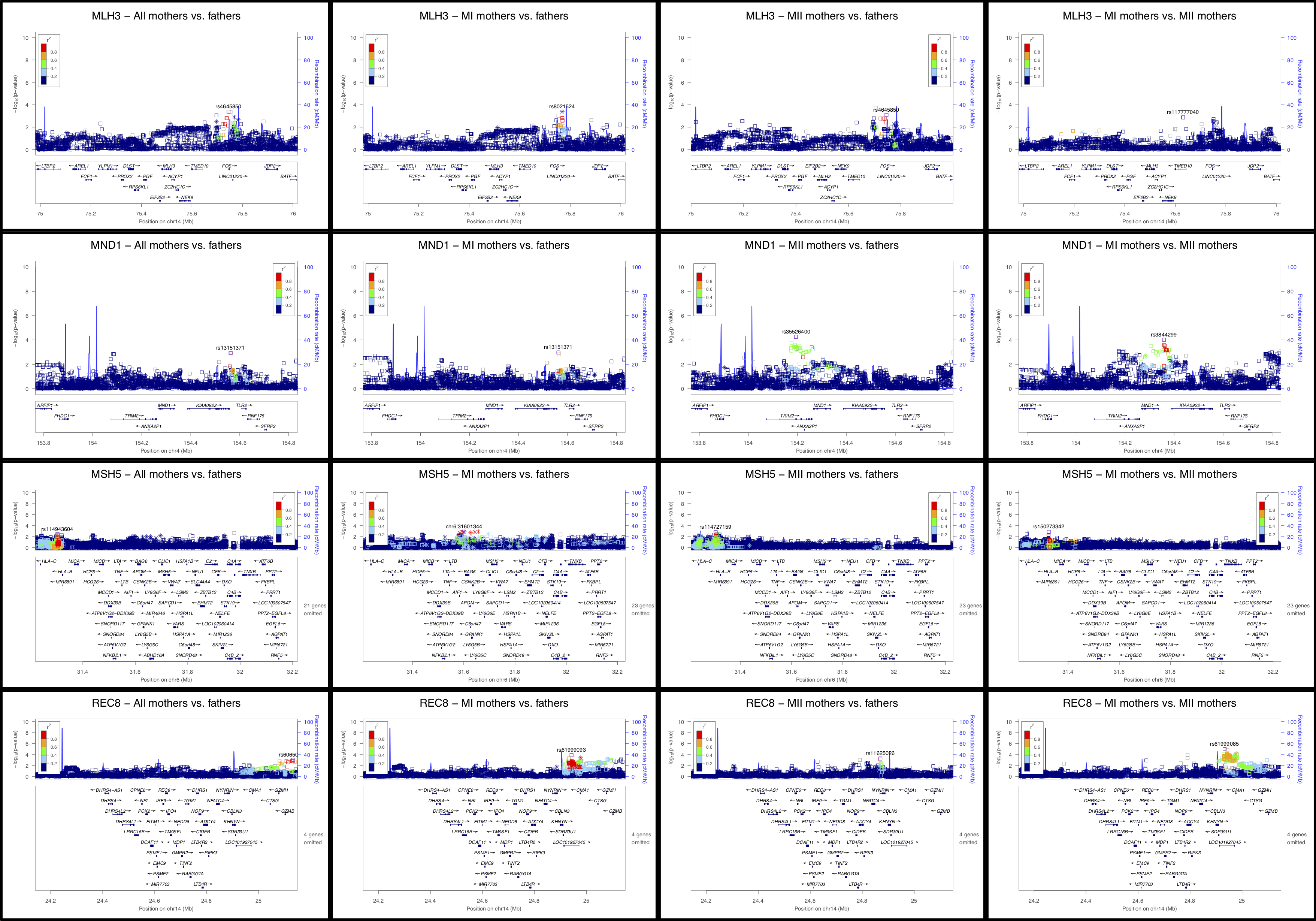

Supplement: S4 Fig — (TIFF) [file pgen.1008414.s004.tiff]

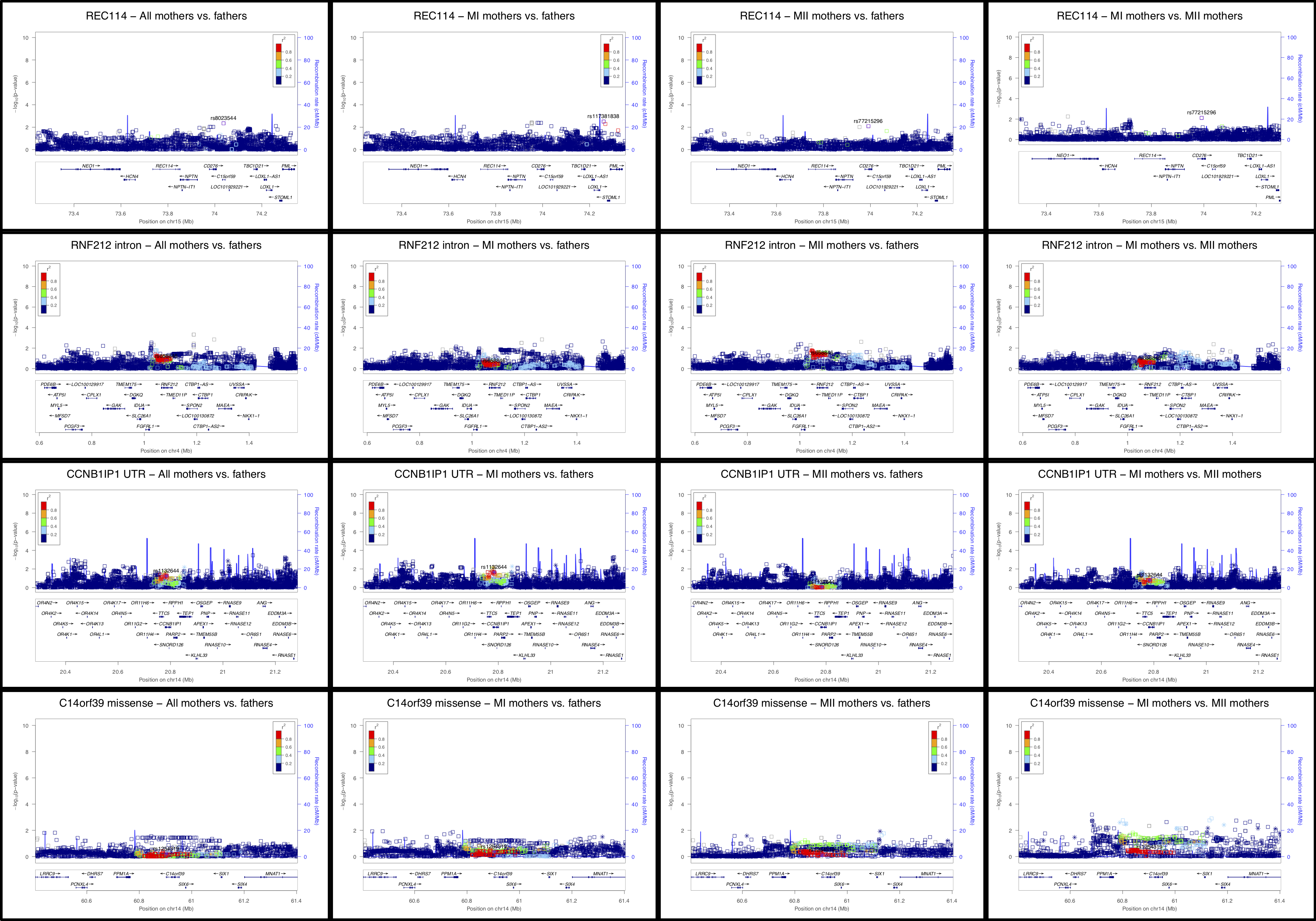

Supplement: S5 Fig — (TIFF) [file pgen.1008414.s005.tiff]

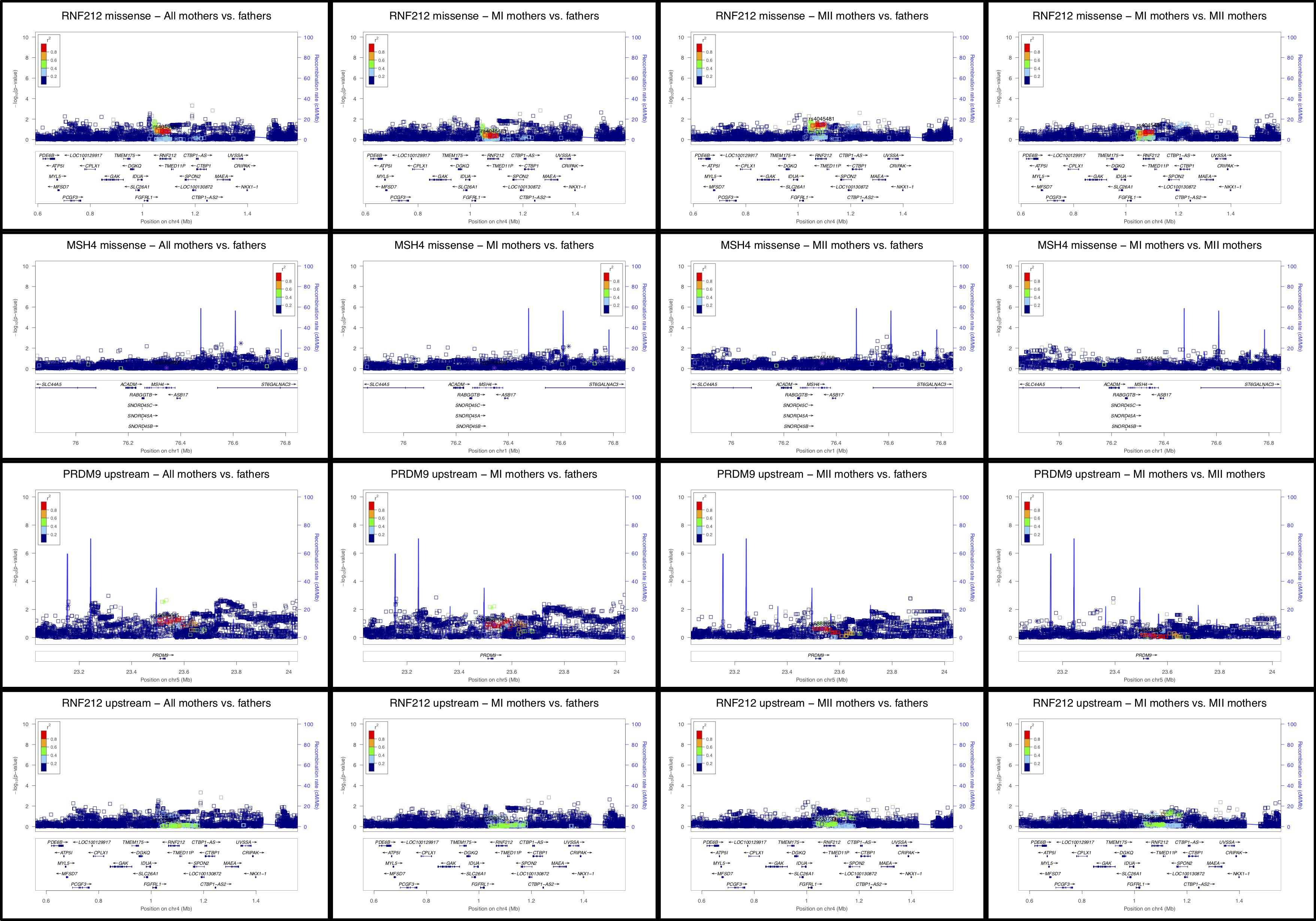

Supplement: S6 Fig — (TIFF) [file pgen.1008414.s006.tiff]

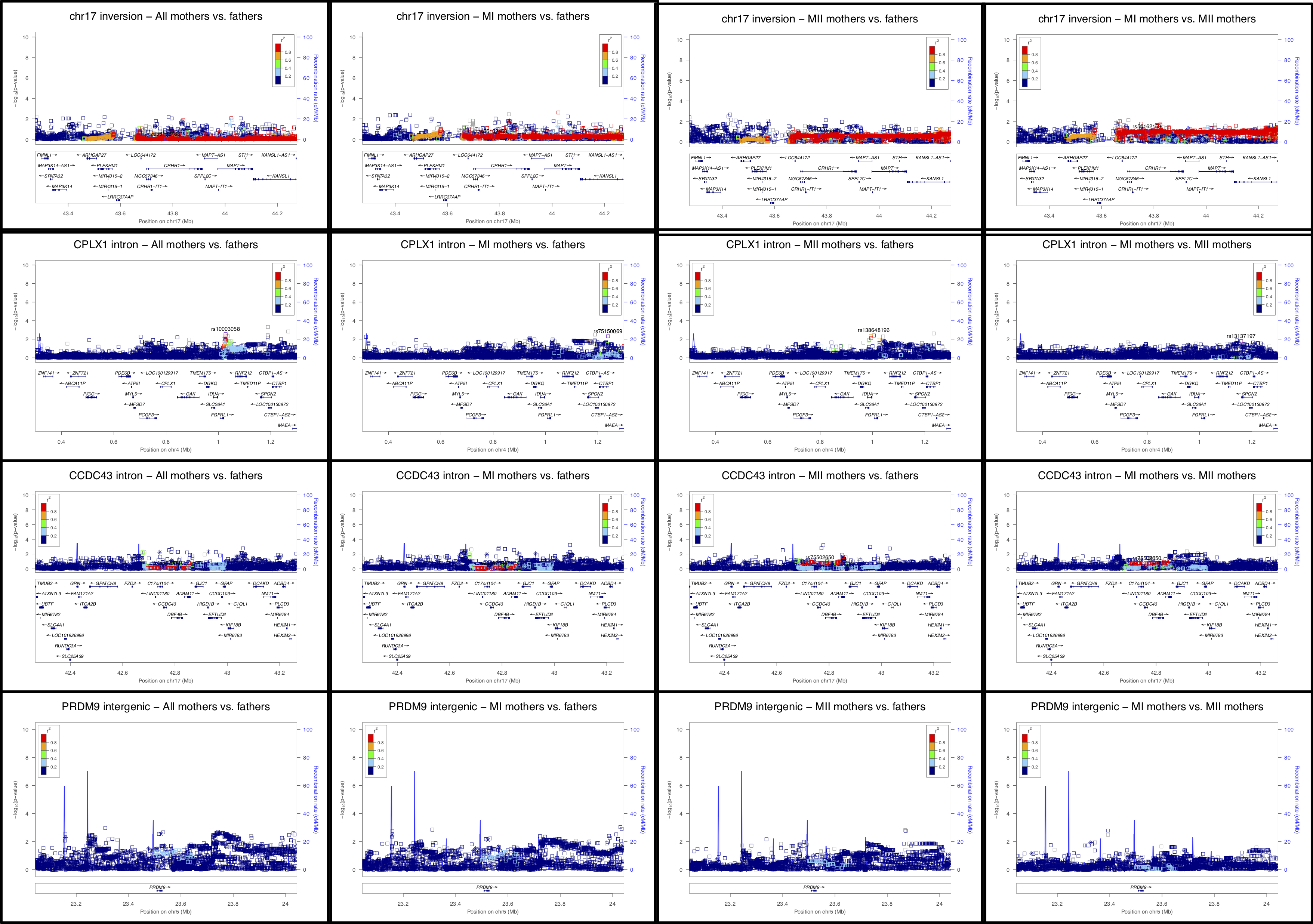

Supplement: S7 Fig — (TIFF) [file pgen.1008414.s007.tiff]

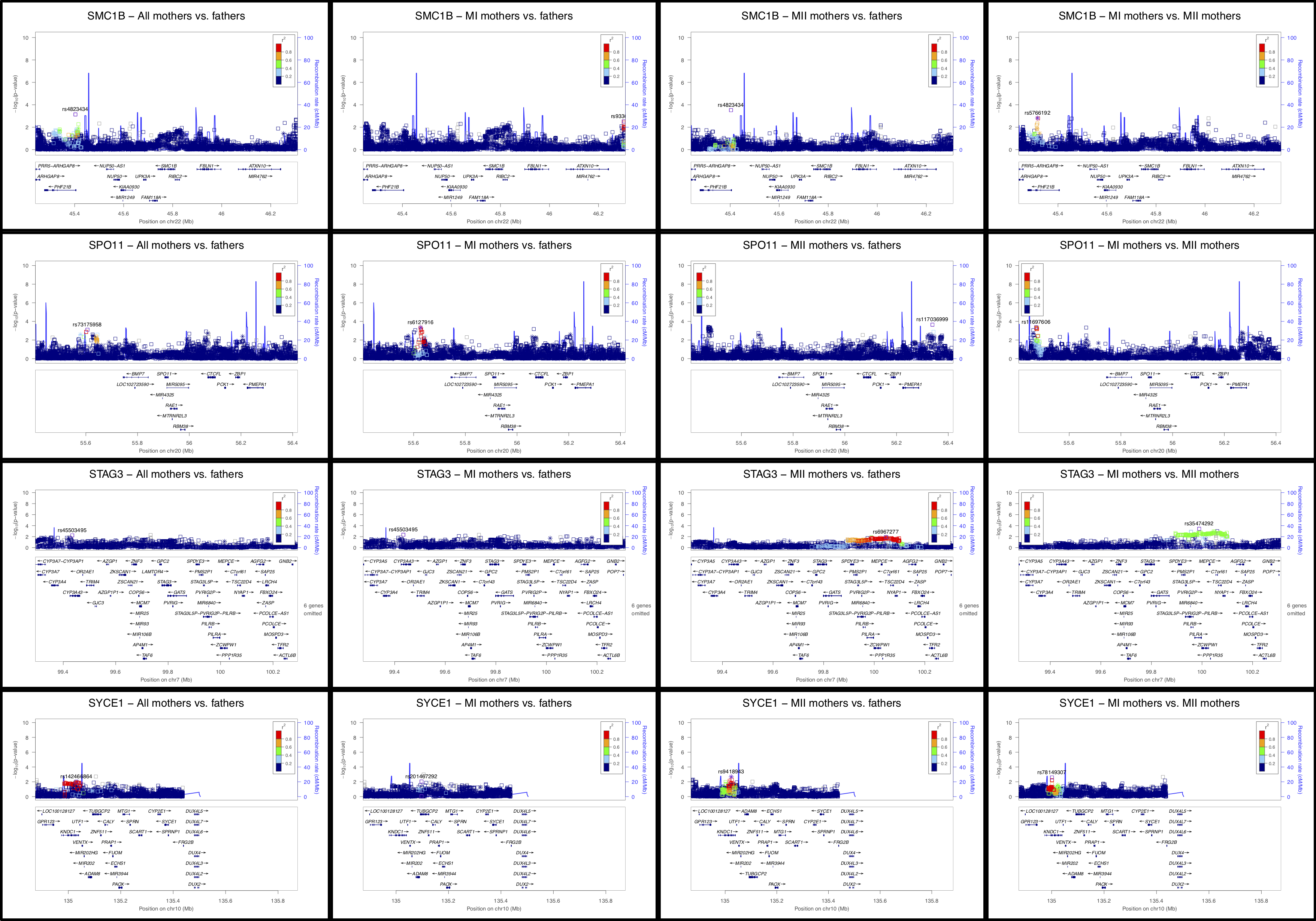

Supplement: S8 Fig — (TIFF) [file pgen.1008414.s008.tiff]

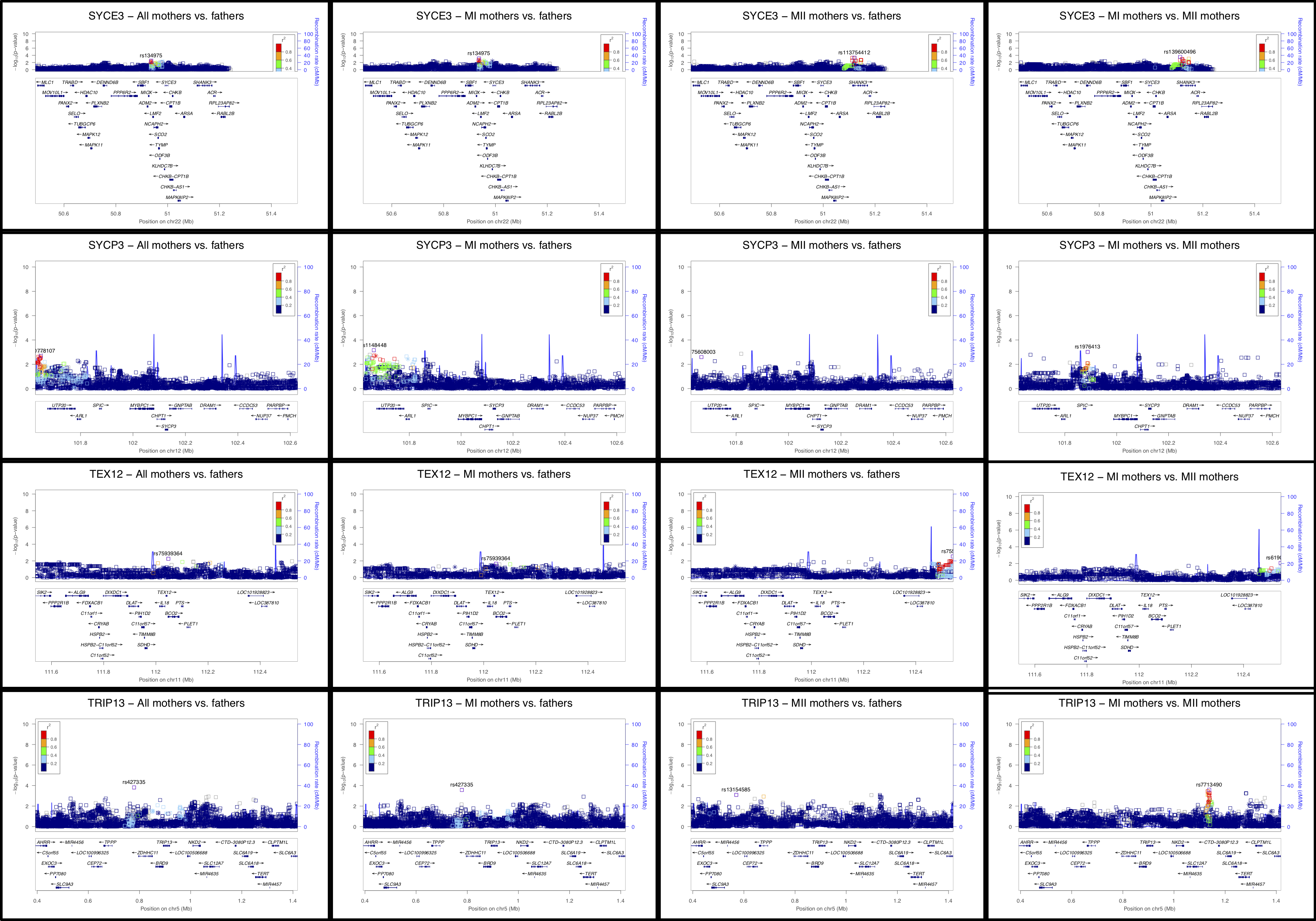

Supplement: S9 Fig — (TIFF) [file pgen.1008414.s009.tiff]

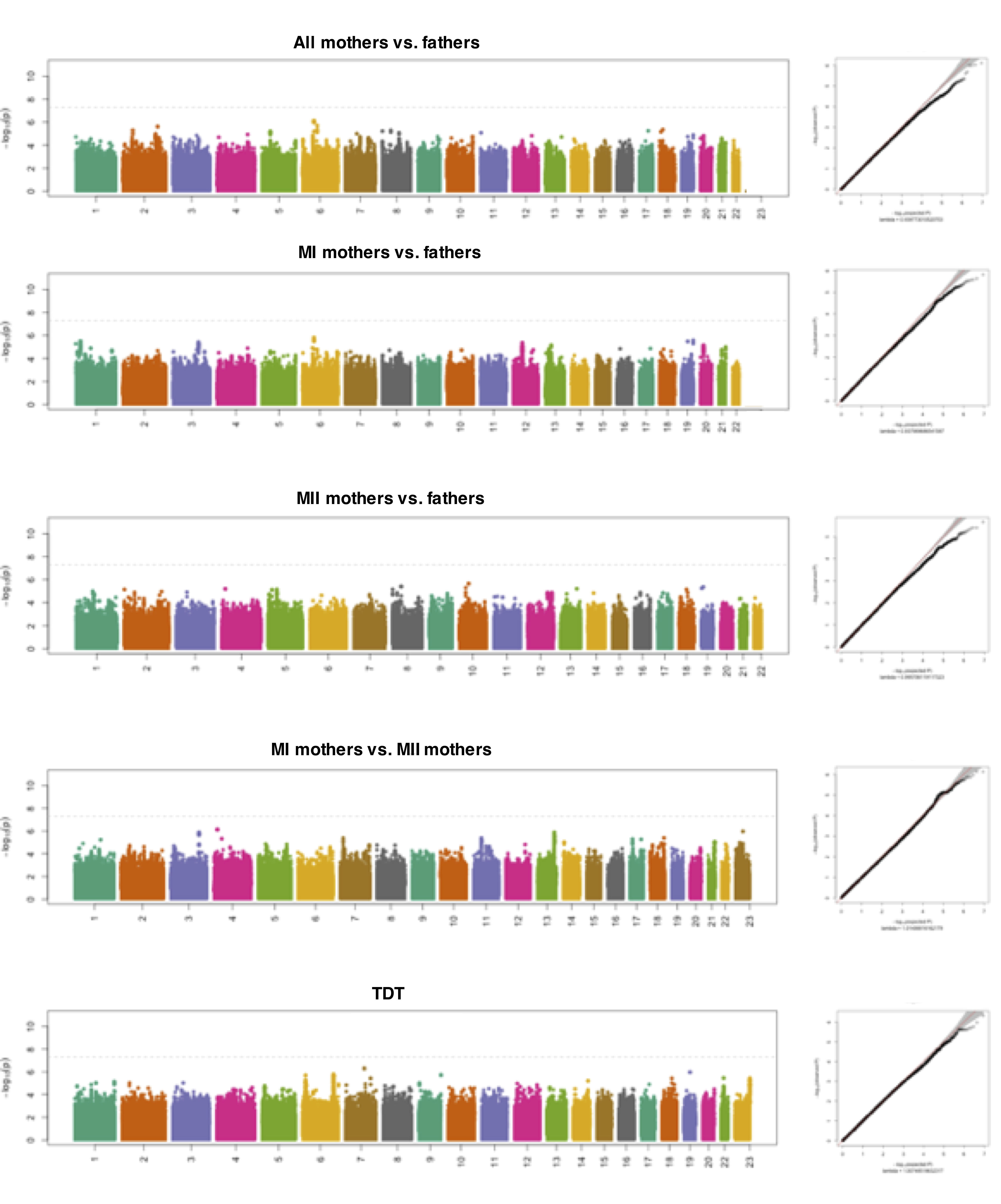

Supplement: S10 Fig — (TIFF) [file pgen.1008414.s010.tiff]

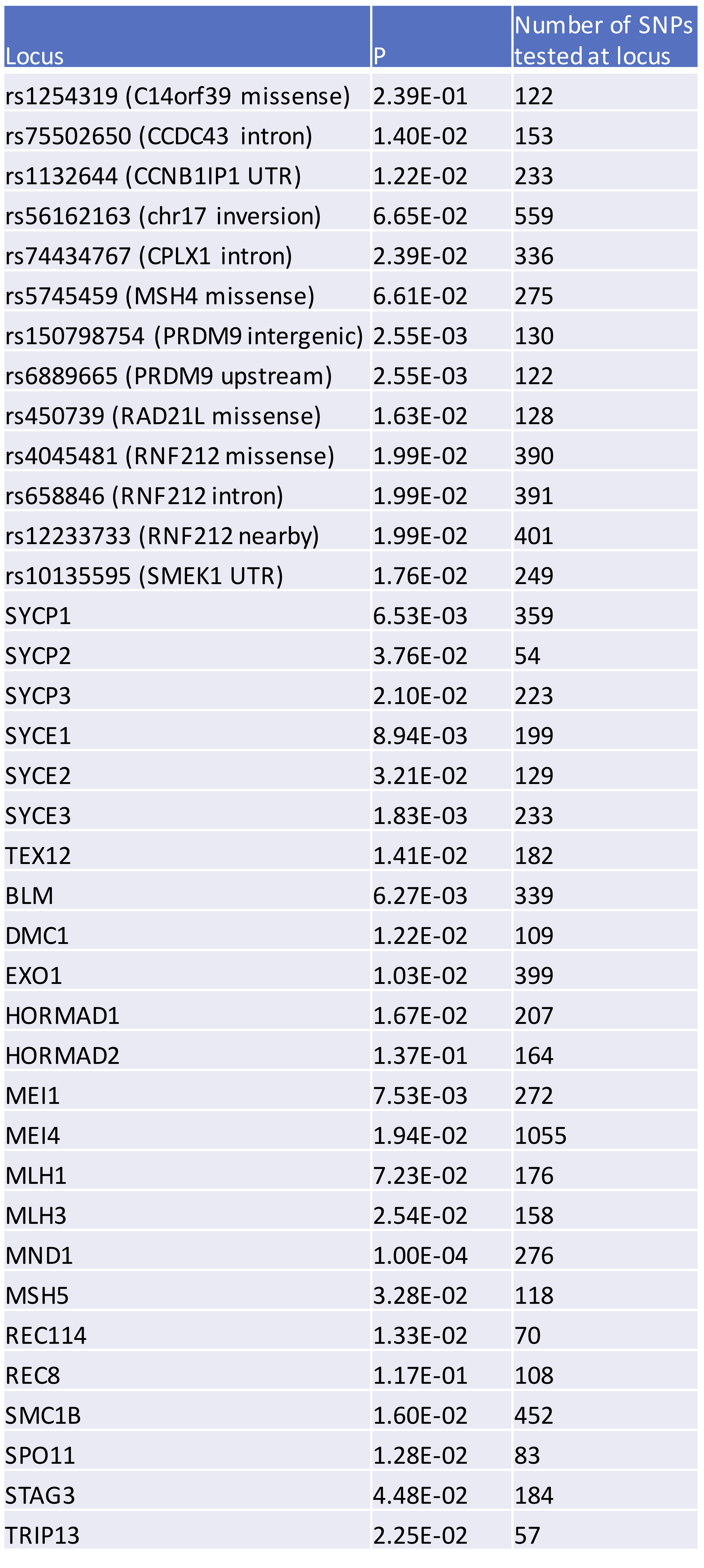

Supplement: S1 Table — (TIFF) [file pgen.1008414.s011.tiff]

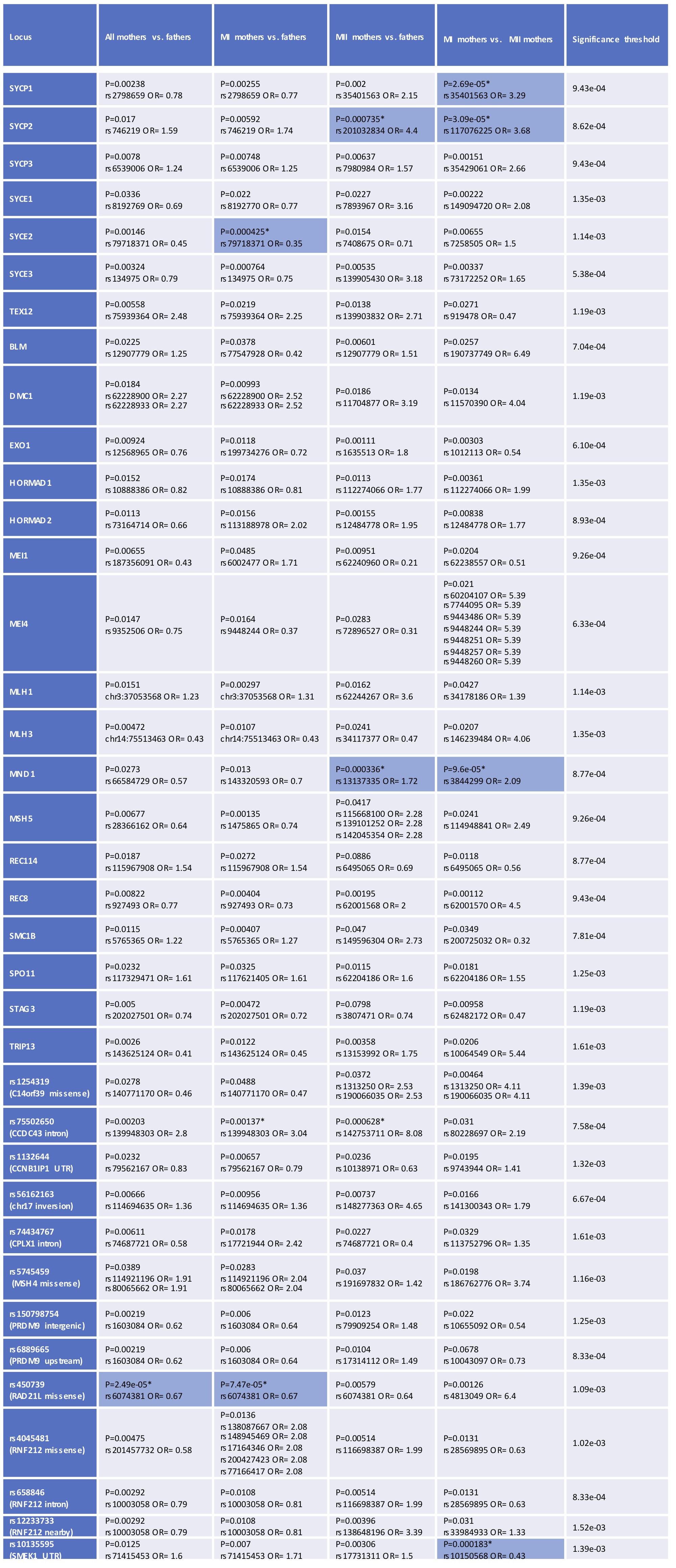

Supplement: S2 Table — Each row represents one candidate locus (either a gene with a 60kb border on each side or a 60kb window around a SNP). Each column represents an analysis. For each locus-analysis pair, the most significant association at the locus (not always unique) is reported. P-values significant after correcting for multiple testing (i.e., exceeding the Bonferroni-adjusted significance threshold noted in the last column) are marked with an asterisk and highlighted. (MI: meiosis I; MII: meiosis II; P: p-value; OR: odds ratio.) The first 24 loci represent genes selected for their function (above the double line). The latter 13 loci represent SNPs identified by Kong et al. in their GWAS of recombination [33], with annotation in parentheses (below the double line). Note that for each analysis in each gene, S2 Table lists the most statistically significant result within a window, so that the SNP that appears in a given gene is not necessarily the same in each analysis. This also means that some odds ratios appear to “flip” between analyses; for example, a result that shows an odds ratio of 2.0 for one SNP in the MI vs. fathers analysis may be represented as an odds ratio of approximately 0.5 (i.e. 1/2.0) for a nearby SNP in the MII vs. fathers analysis. (TIFF) [file pgen.1008414.s012.tiff]

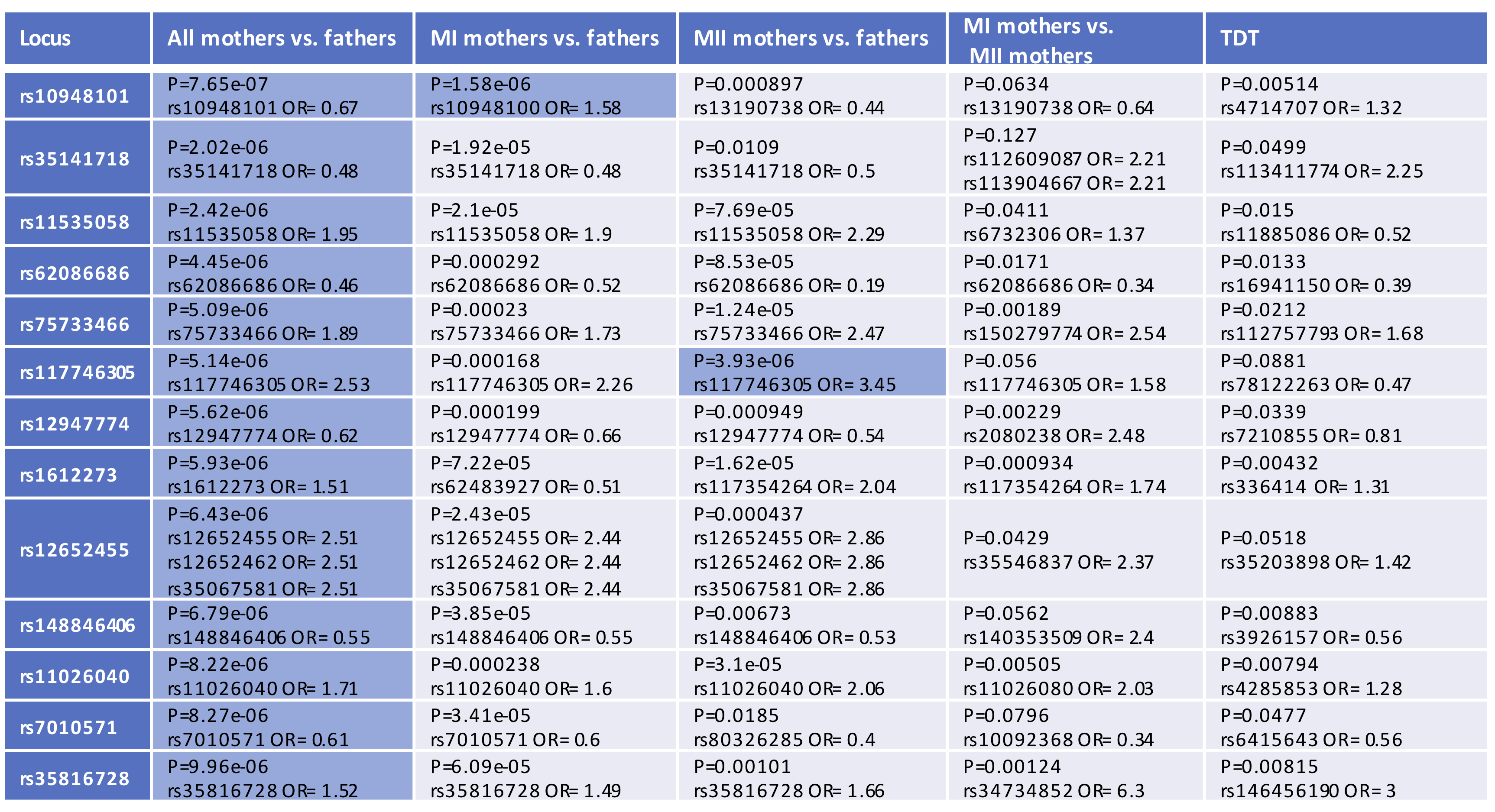

Supplement: S3 Table — In this table (and in S4–S7 Tables) suggestive associations (p < 10−5) are recorded (highlighted cells). For each such locus, the most significant association within 20kb is recorded for each of the other four genome-wide analyses. Rows are ordered by significance. (MI: meiosis I; MII: meiosis II; P: p-value; OR: odds ratio). (TIFF) [file pgen.1008414.s013.tiff]

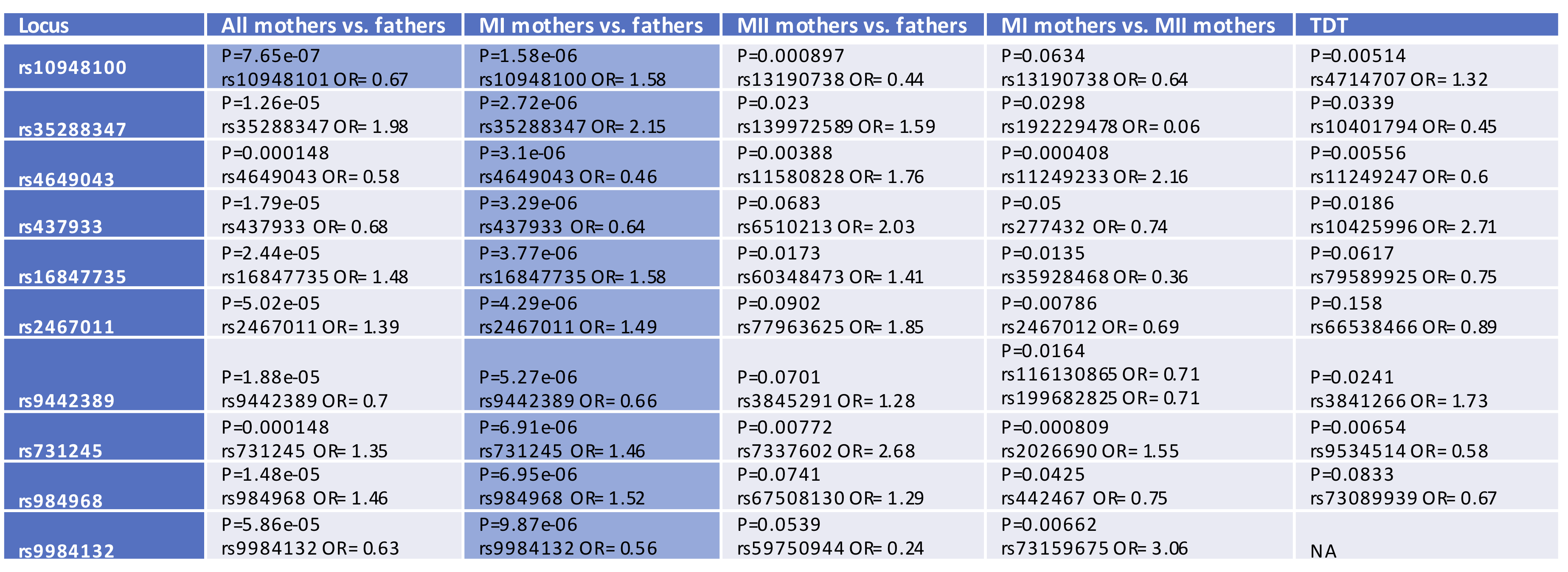

Supplement: S4 Table — (TIFF) [file pgen.1008414.s014.tiff]

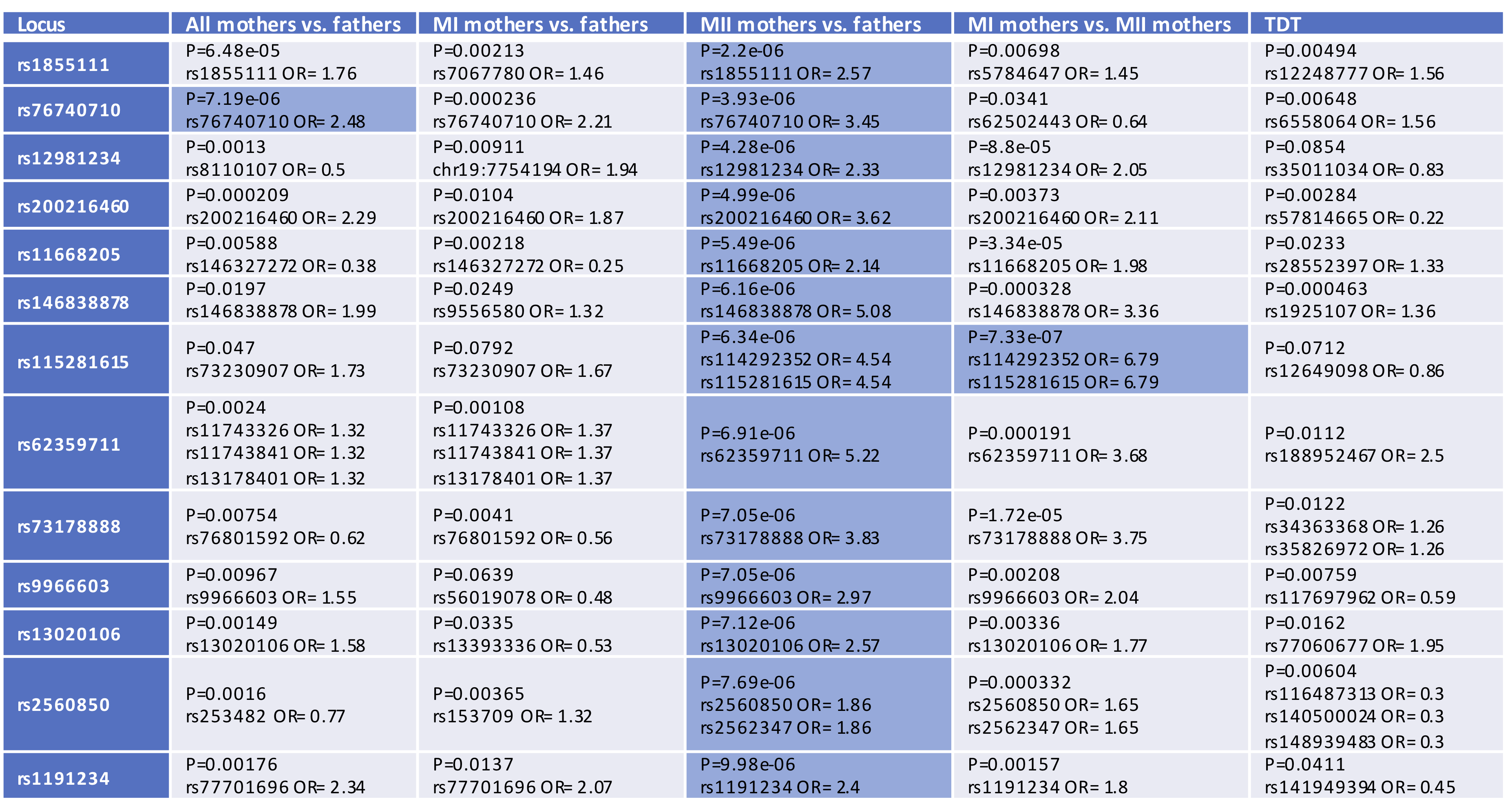

Supplement: S5 Table — (TIFF) [file pgen.1008414.s015.tiff]

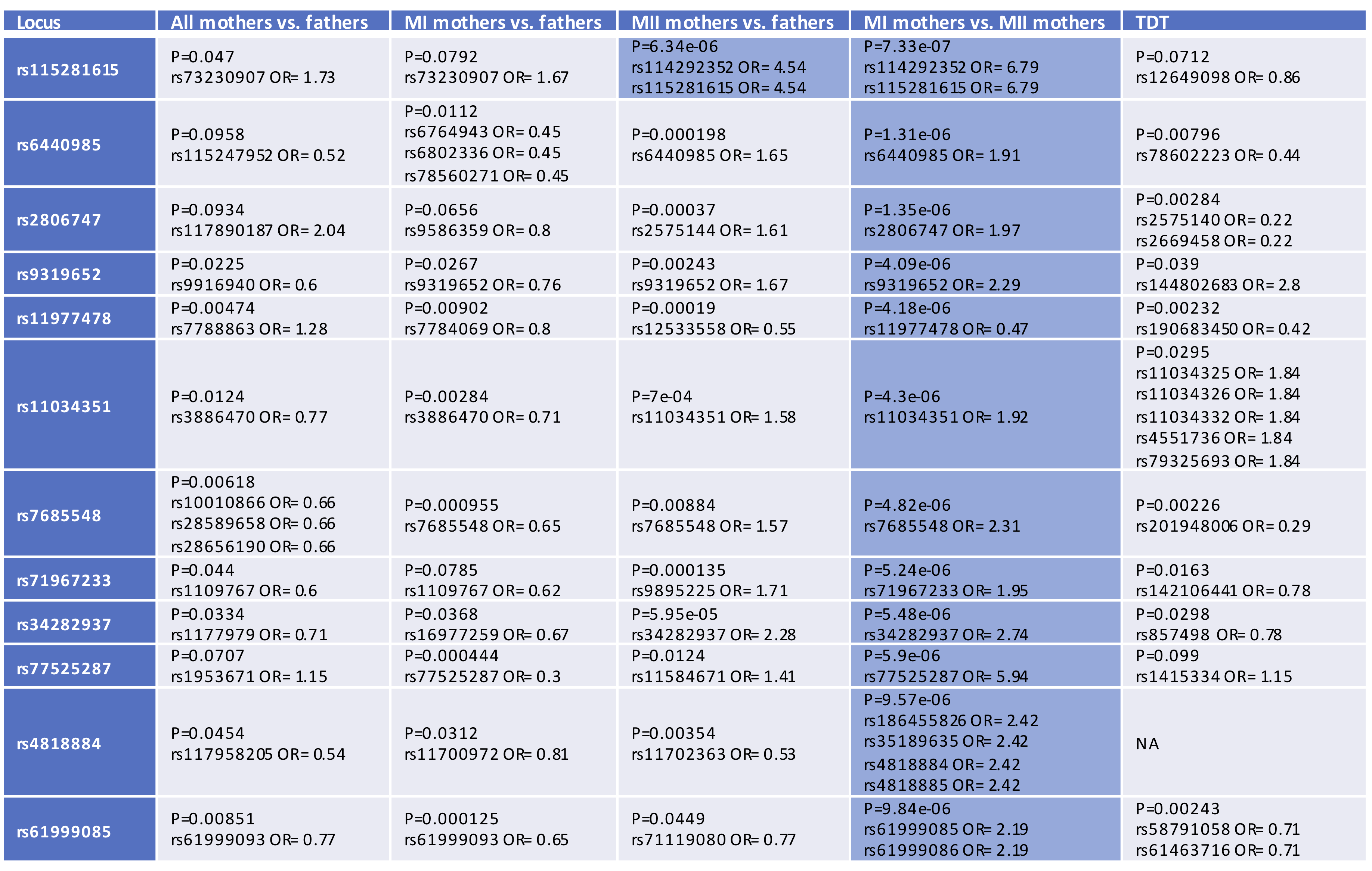

Supplement: S6 Table — (TIFF) [file pgen.1008414.s016.tiff]

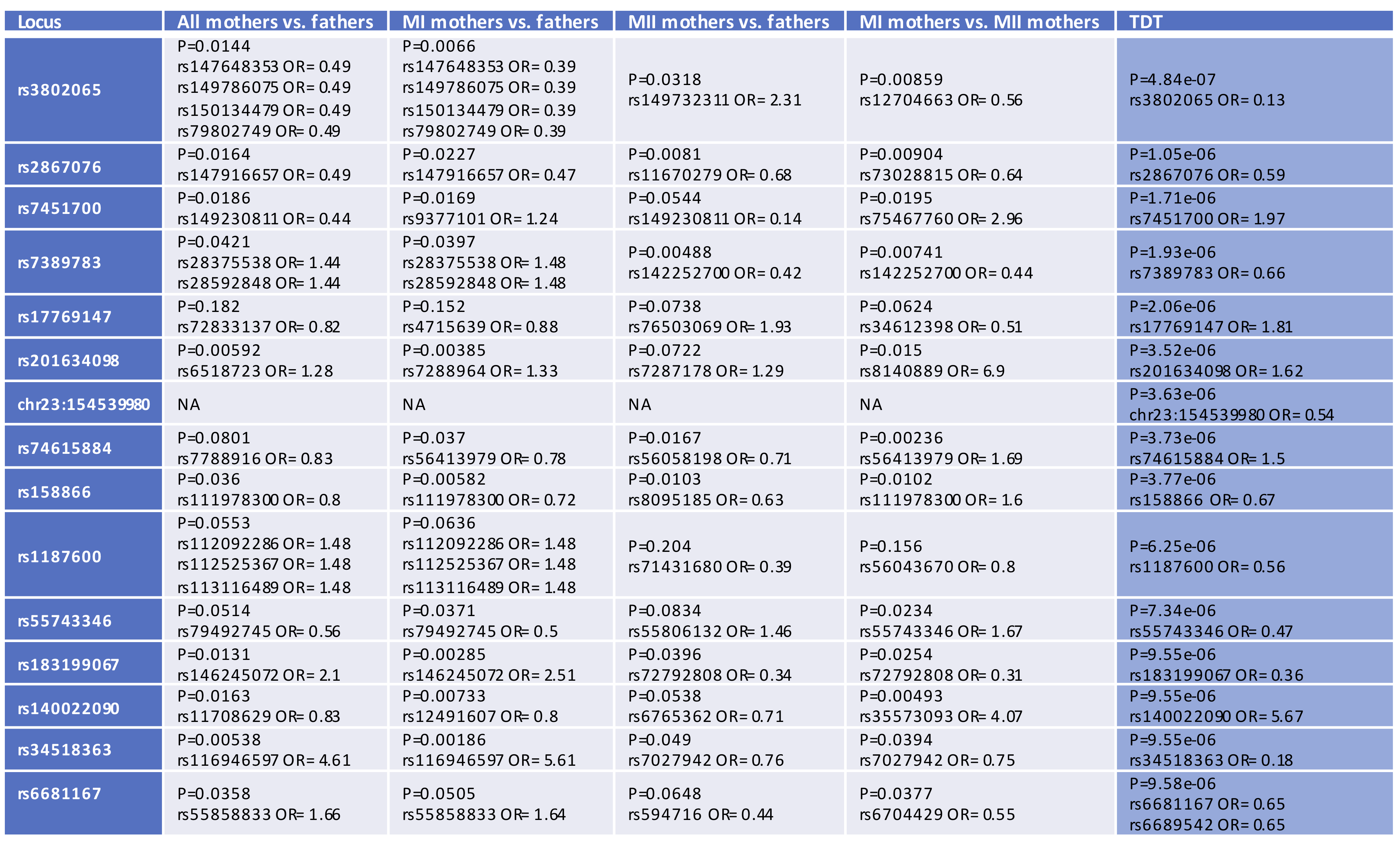

Supplement: S7 Table — (TIFF) [file pgen.1008414.s017.tiff]
